# An Embodied Agent Learning Affordances With Intrinsic Motivations and Solving Extrinsic Tasks With Attention and One-Step Planning

**DOI:** 10.3389/fnbot.2019.00045

**Published:** 2019-07-26

**Authors:** Gianluca Baldassarre, William Lord, Giovanni Granato, Vieri Giuliano Santucci

**Affiliations:** ^1^Laboratory of Computational Embodied Neuroscience, Institute of Cognitive Sciences and Technologies, National Research Council of Italy, Rome, Italy; ^2^School of Engineering Sciences, KTH Royal Institute of Technology, Stockholm, Sweden

**Keywords:** open-ended learning, intrinsic motivations, affordance learning, goal-based planning, utility-based planning, active-vision, attention

## Abstract

We propose an architecture for the open-ended learning and control of embodied agents. The architecture learns action affordances and forward models based on intrinsic motivations and can later use the acquired knowledge to solve extrinsic tasks by decomposing them into sub-tasks, each solved with one-step planning. An affordance is here operationalized as the agent's estimate of the probability of success of an action performed on a given object. The focus of the work is on the overall architecture while single sensorimotor components are simplified. A key element of the architecture is the use of “active vision” that plays two functions, namely to focus on single objects and to factorize visual information into the object appearance and object position. These processes serve both the acquisition and use of object-related affordances, and the decomposition of extrinsic goals (tasks) into multiple sub-goals (sub-tasks). The architecture gives novel contributions on three problems: (a) the learning of affordances based on intrinsic motivations; (b) the use of active vision to decompose complex extrinsic tasks; (c) the possible role of affordances within planning systems endowed with models of the world. The architecture is tested in a simulated stylized 2D scenario in which objects need to be moved or “manipulated” in order to accomplish new desired overall configurations of the objects (extrinsic goals). The results show the utility of using intrinsic motivations to support affordance learning; the utility of active vision to solve composite tasks; and the possible utility of affordances for solving utility-based planning problems.

## 1. Introduction

**Figure 1:**
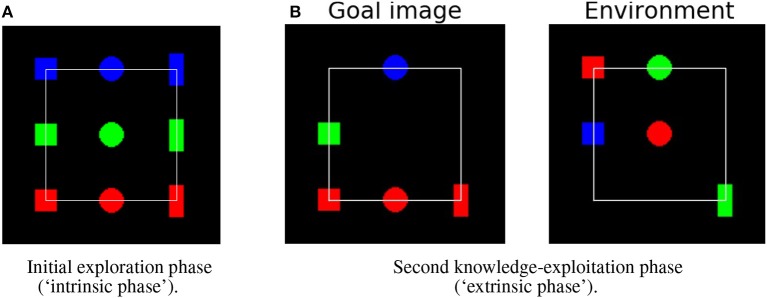
Example scenario used to test the open-ended learning architecture proposed here. **(A)** Intrinsic phase: initial configuration of the environment containing nine 2D “objects.” Each object has a certain position and color (the “object state”). In this phase the agent can autonomously explore the objects for a certain time to acquire as much knowledge and skills as possible. **(B)** Extrinsic phase: example of a task that the agent has to solve, requiring to either move objects or change their colors to bring the “environment” to the state of the “goal image”; the agent has to be able to do this on the basis of the knowledge and skills acquired during the intrinsic phase. Note that in these images the objects are set on the vertices of a 3 × 3 regular grid, although during exploration and extrinsic-task solving they can occupy any non-overlapping position with their center within the white square frame.

This work proposes an architecture for the control and learning of embodied agents. The architecture has been developed within an open-ended learning context. Figure 1 shows a typical scenario used in such a context^1^: the scenario is used here to test the proposed architecture. The general structure of the scenario involves two phases (Baldassarre, 2011; Seepanomwan et al., 2017): (a) a first *intrinsic motivation phase* where the agent is not given any task and should freely explore the environment to autonomously acquire as much general-purpose knowledge as possible; (b) a second *extrinsic motivation phase* where the agent has to solve one or more tasks assigned externally within the same environment (*extrinsic tasks*). Importantly, the extrinsic phase can furnish an objective measure of the quality of the algorithms used by the agent to autonomously learn during the intrinsic phase. In the intrinsic phase of the specific scenario used here, the agent can perceive objects and explore and learn the effects of certain pre-wired actions (e.g., “move in space” or “change object color”). In the extrinsic phase, the agent is required to use the knowledge acquired in the intrinsic phase to solve extrinsic tasks: first the agent has to memorize the state of some objects set in a certain configuration (*goal*; notice how this is a handy way to allow the agent to store the goal in a format suitable for its processes); then the objects are “shuffled” into a different state (“initial state”); last the agent has to bring the objects back to the goal state.

**Figure 2:**
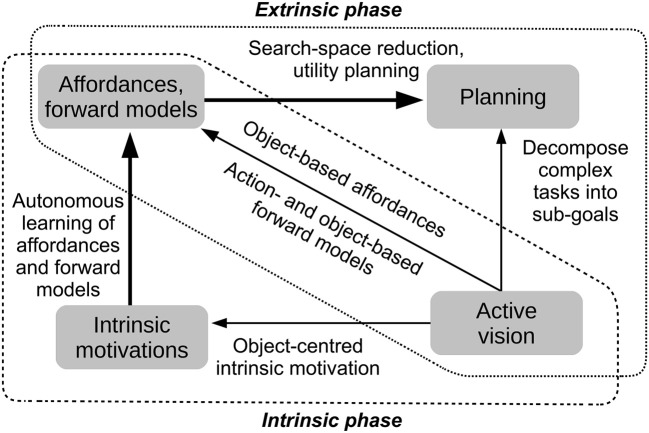
Main functions (gray boxes) and their relations (arrows with labels) implemented by the architecture proposed here. The dashed and dotted frames contain the main functions used in the intrinsic phase and extrinsic phase, respectively. During the intrinsic phase *intrinsic motivations* support the autonomous learning of *affordances and forward models*; during the extrinsic phase, *affordances and forward models* support *planning* to accomplish extrinsic tasks (bold arrows). Importantly, in the two phases *active vision* supports these processes (thin arrows): to generate intrinsic motivations linked to objects; to learn affordances and forward models related to objects; and to plan based on parsing the goal into object-related sub-goals.

Facing the challenges posed by the scenario requires different functions. The functions used by the architecture proposed here are summarized in Figure 2. The figure shows that during the intrinsic phase the architecture uses intrinsic motivations to learn action affordances and forward models, and during the extrinsic phase it uses affordances and forward models to plan and solve the extrinsic tasks. Importantly, in both phases active vision allows the agent to focus on a single object per time, in particular to elicit object-centered intrinsic motivations, to learn or activate the affordances and the forward models related to specific objects, and to parse the extrinsic goal into simpler sub-goals each achievable with 1-step planning. These processes are now considered more in detail. For each process we now highlight the relevant concepts and literature and introduce the open problems faced here (section 4 compares the architecture with other specific models proposed in the literature). We then illustrate the three main contributions of the work.

### 1.1. Links to the Literature and Open Problems

The proposed architecture has been developed within the area of developmental and autonomous robotics called *open-ended learning* (Thrun and Mitchell, 1995; Weng et al., 2001). Open-ended learning processes allow robots to acquire knowledge (e.g., goals, action policies, forward models, and inverse models, etc.) in an incremental fashion by interacting with the environment. These learning processes are strongly inspired by the exploration processes seen in animals, in particular in humans and especially in children (Asada et al., 2001; Lungarella et al., 2003). Although open-ended learning processes can involve both social and individual mechanisms (Baldassarre and Mirolli, 2013a), here we only focus on individual learning processes supporting an autonomous acquisition of knowledge.

A central concept in open-ended learning is the one of *intrinsic motivations* (IMs). These are mechanisms for driving autonomous learning (White, 1959; Ryan and Deci, 2000; Oudeyer and Kaplan, 2007; Baldassarre and Mirolli, 2013a). The utility and adaptive function of IMs reside in that they can produce learning signals, or trigger the performance of behaviors, to drive the acquisition of knowledge and skills that become useful only in later stages with respect to the time in which they are acquired (Baldassarre, 2011). There are various IM mechanisms, some based on the *novelty* or *surprise* of the stimuli (Baldassarre and Mirolli, 2013b; Barto et al., 2013), and others based on the agent's *competence*, i.e., its capacity to successfully accomplish a desired outcome (Mirolli and Baldassarre, 2013).

While various works use IMs as a means to directly guide the autonomous learning of skills (e.g., Schmidhuber, 1991b; Oudeyer et al., 2007), recently they have been used in connection to goals. In particular, surprise or novelty IMs can be used to generate goals, and the goal accomplishment rate can be used to measure competence (Barto et al., 2004; Santucci et al., 2012, 2016; Baranes and Oudeyer, 2013). By *goal* here we refer to an internal representation of the agent having the following properties (cf. Fikes and Nilsson, 1972; Bratman, 1987): (a) the representation refers to a possible future state/state-trajectory (or set of states/state-trajectories) of the world; (b) the representation can be re-activated on the basis of internal processes in the absence of that state/state-trajectory in the world; (c) if the agent “activates”/“commits” to the goal, the goal motivates the performance of some behaviors, in particular the performance of behaviors that tend to push the environment toward the goal state; (d) when the environment reaches (or is close to) the goal state, learning signals or motivations to act in a certain way can be generated; (e) a sub-goal is a goal that is not pursued *per se* but as a means to achieve a desired “final” goal. The architecture presented here, as usually done in the robotic literature on affordances (see below), assumes the agent is given a set of actions and the capacity to recognize if the performance of those actions leads to their desired effects (goals): the challenge for the robot is indeed to use intrinsic motivations to learn which actions can be successfully accomplished on which objects (object affordances).

Much research on open-ended learning has focused on the autonomous acquisition of knowledge during “intrinsically motivated” phases. On the other side, only a few works (e.g., Schembri et al., 2007; Santucci et al., 2014; Seepanomwan et al., 2017) have focused on how such knowledge can be exploited later to solve “extrinsic tasks,” namely tasks that produce a material utility to the agent (or to its user in the case of robots, Baldassarre, 2011). The work presented here focuses on problems involving both the intrinsic and extrinsic phases. Although the intrinsic and extrinsic learning processes are often intermixed in realistic situations, separating them can help to clarify problems and to develop algorithms, most notably to use the performance in the extrinsic phase to measure the quality of the knowledge autonomously acquired in the intrinsic phase.

In the extrinsic phase, we consider a test that requires solving a complex task formed by multiple sub-tasks each involving a specific object. The reason why we focus on these types of complex tasks is that: (a) they are involved in most sensorimotor non-navigation robotics scenarios requiring object-manipulation; (b) active perception (see below) can be extremely useful to tackle these scenarios. A possible strategy to solve complex tasks is based on *planning* (Ghallab et al., 2004), directed to assemble sequences of sub-goals/skills leading to accomplishing the overall complex goal. In this work we focus on a class of tasks where the sub-tasks are (cf. Korf, 1985; Russell and Norvig, 2016): (a) *independent* between them, meaning that the accomplishment of one of them does not require the previous accomplishment of another; (b) *serializable*, meaning that the accomplishment of each sub-goal does not violate other already accomplished sub-goals; (c) can be achieved with a single action available to the agent. An example of such a task in the domain considered here, involving two sub-goals, is: “turn the red circle into a blue circle and move the red square to a desired x-y position.” This type of task can be solved with repeated single-step planning processes. The use of this simple class of problems allows us to focus on the issues illustrated below.

In order to study the relations between affordances (see below) and planning, we focus here on two types of planning strategies investigated in the literature on planning (Ghallab et al., 2004; Russell and Norvig, 2016). The first type involves *goal-based planners* that have to decide which actions to perform to accomplish a desired future condition (goal). The second type involves *utility-based planners* that have to decide between alternative conflicting goals having a different desirability and pursued in uncertain conditions (here the stochasticity of the environment is due to the fact that actions succeed only with a certain probability).

As mentioned above, an important dimension of the focus of this work concerns *affordances*. This concept was first proposed within the psychological literature to refer to the actions that a given condition or object “offers” to an agent given its current body state (Gibson, 1979). The concept has been later used in the developmental-robotics literature to refer to the dyadic relational concept for which a certain condition or *object* allows performing a certain *action* on it (Stoytchev, 2005; Sweeney and Grupen, 2007). The interest of affordances for autonomous robotics, as we will also show here, resides in the fact that they can represent a simple and efficient mechanism to rapidly decide which actions can be performed, with some potential utility, on the objects available in the environment.

In the literature, the concept of affordance has occasionally been broadened to refer to various elements relevant for planning. For example, affordances have been associated with three functions linking three critical elements of behavior (Montesano et al., 2008; Ugur et al., 2011): an *Object* (*O*), the *Action* (*A*) to perform on it, and the resulting *Effect* (*E*). The three functions, denoted here as *F*, *C* and *I*, get as input two of the three elements and return the remaining one as output: (a) forward model, *E* = *F*(*O, A*); (b) relevance, *O* = *C*(*A, E*); (c) inverse model, *A* = *I*(*O, E*). With respect to planning, these functions can play various roles. “Forward models” can be used to support forward planning, as here, because they allow the agent to predict the effect of performing a given action *A* on a certain object *O* and check if the obtained effect *E* matches a desired goal/sub-goal (*G*). “Relevance” checking (Russell and Norvig, 2016) allows the agent to search actions for which the effect fulfills the goal and then to search for relevant objects (on which the actions can be applied) to accomplish the goal (the function *C* has also been used often in the affordance literature to perform sheer object recognition based on how objects respond to actions, e.g., see Fitzpatrick et al., 2003; Castellini et al., 2011; Nguyen et al., 2013; Ugur and Piater, 2014). “Inverse models” can be used to directly perform actions, with the caveat that the object does not represent the whole state of the environment as required by proper inverse models (this is not further discussed here). These broad definitions of affordance are important to highlight the triadic relational nature of affordances, involving not only *object/conditions* and *actions* but also *action effects*. On the other hand, such definitions overlap to a certain degree with other concepts used in the computational literature and this decreases their utility.

Here we contribute to the investigation of the possible functions of affordances for autonomous agents by assuming a restricted definition of them. This definition allows us to evaluate the utility of affordances within planning systems and also to contribute to clarifying the relation between the concept of affordance used in psychology and in robotics. Informally, the definition is this: *an affordance is an agent's estimated probability that a certain action performed on a certain object successfully accomplishes the desired outcome associated with the action (“goal”)*. Formally, the definition of affordance used in this work is as follows:

*An affordance is an agent's estimated probability* $Pr\left(s_{b,o}^{\prime }\in G|a,s_{b,o}\right)$ *that if it performs action a on the object o when the object and own body b are in state s_b,o_ then the outcome will be a state* $s_{b,o}^{\prime }$ *belonging to a set of goal states G to which the action is directed*.

We illustrate the features of this definition and its differences and links with other definitions. (a) The definition is more specific than other definitions that often have vague features. (b) The states *s*_*b, o*_ refer to a certain object and the agent's body so the definition is closely linked to the original idea of affordance as founded on the body-object relation. Moreover, the focus on “b” and “o” differentiates the concept from the *transition models* (linking current state and action to future states) used in the reinforcement learning literature (Sutton and Barto, 2018) as these use *atomic/whole-state representations*; instead, such reference links the definition to the *preconditions* used in symbolic planning operators that use *structured representations* capturing the relations between different entities (here “o” and “b”; Russell and Norvig, 2016). (c) The definition is centered on the concept of “object,” intended here as a limited portion of matter that is physically detached from the rest to the environment. This is very important as in the literature on affordances objects are crucial for *manipulation tasks*, an important class of robotic tasks alongside *navigation tasks*. As discussed below, the focus on objects makes attentional processes very useful for the acquisition and use of affordances. (d) Importantly, the definition is grounded on the link between the action and its *goal*, i.e., the possible effects that the action is expected to produce on the object; for example, a “grasping action” might have the goal “hand envelops the object.” This is important as often in the computational literature on affordances it is assumed that the agent is able to check the success of an action performed on a certain object (e.g., that “the object rolls” if pushed) without fully recognizing that such a check needs a reference state/event with which to compare the action outcome; that is, it needs a goal. (e) The goal can be abstract in the sense that it encompasses different states *s*_*b, o*_. In particular, it can be abstract with respect to the elements of the environment other than the object and body. Sometimes the goal might even be abstract with respect to the body features; for example different robots with different actuators could all be able to “grasp” a certain object. In some cases, the goal might abstract altogether from the body and involve more than one object, in particular some relation between them; for example the goal might require piling “object A on object B” (in this case the affordance is that A is “pileable” on B). The “o” in the definition might even go beyond “objects” and include broader affordances; for example Ugur et al. (2007) studied the “traversability” of a portion of environment in a navigation task. (f) There can be many different possible goals *G*, and actions to pursue them, that an agent can perform on a given object; for example a robot can “push,” “grasp,” or “lift” a given object to accomplish different goals. (g) The definition, based on a probability, takes into account the uncertain nature of the environment where the performance of actions does not necessarily produce the desired outcomes. (h) The definition assumes a binary success/failure of the action, for example based on the use of a threshold (e.g.: “the object is considered as reached if the distance between the object and the hand-palm after action execution is smaller than 2 cm”). An alternative definition, assuming that suitable distance metrics could be applied to the object/body states, could state that the affordance is accomplished *in a continuous degree* related to the final distance of the state achieved by the action and the reference goal-state (e.g.: “a reaching action toward an object brought the hand 5 cm close to it”). Here we will use the first definition related to probability-based binary affordances. Within this, we will consider two types of affordances. The first, called here *deterministic affordances*, where objects *allow or not* the agent to accomplish the goal (e.g., an object could be “movable” or “non-movable”): in this case the affordance probability is equal to 0 or 1. The second, called here *stochastic affordances*, can accomplish the goal only with a certain probability, for example an object might be “movable” with a probability of 0.7 and another one with a probability of 0.3.

We now introduce a pivotal feature of our system, the use of *active vision* (Ballard, 1991; Ognibene and Baldassare, 2015). Agents with active vision: (a) use a visual sensor that returns information related to only a limited portion of the scene; (b) actively direct the sensor on relevant regions of space (“overt attention”). These assumptions reflect fundamental principles of organization of the visual system of primates (Ungerleider and Haxby, 1994) and in artificial systems they allow the reduction of visual information processing and an easier analysis of spatial relations between scene elements (Ognibene and Baldassare, 2015). Here active vision is used to extract information on objects, very important for three processes: intrinsic motivations, affordance processing, and planning. Regarding intrinsic motivations, we shall see that our system relates them to objects and on this basis decides on which object to invest exploration and learning. Regarding affordances, we have seen above that most works in the literature use setups structured so that the system can gather information on affordances related to *single objects* (e.g., see Fitzpatrick et al., 2003). The system proposed here uses active vision to focus on single objects and detect their affordances. Regarding planning, the classic AI planners usually employ *structured knowledge representations* of states and actions (“operators”) expressed with propositional logic or first order logic; this type of representation is very important as it allows systems to “reason” about objects and their relations, and “almost everything that humans express in natural language concerns objects and their relationships” (Russell and Norvig, 2016). For our focus on embodied systems, we instead use here *factored knowledge representations* based on feature vectors, in particular image features. However, active vision allows the system to identify single objects, and this information allows the system to parse overall goal images into object-related sub-goals that can be pursued one by one. The planning processes considered here are akin to those used within the *Dyna systems* of reinforcement learning literature where planning is implemented as a reinforcement learning process running within a world model rather in the actual environment (Sutton, 1990; Baldassarre, 2002; Sutton and Barto, 2018). Some caveats on the approach used here to visually isolate objects are due. We simplify the task by not considering cluttered scenarios. Moreover, we use simple bottom-up attention processes to control gaze. These simplifications allow us to develop the overall architecture of the system, but in future work some of the components of the system could be substituted by more sophisticated components, in particular for object segmentation and detection (Yilmaz et al., 2006; Zhang et al., 2008; LeCun et al., 2015) and for a smarter “top-down” control of gaze depending on the agent's information needs (Ognibene et al., 2008, 2010; Dauce, 2018).

### 1.2. Contributions of the Work

A first contribution of this study is on how intrinsic motivations can support efficient learning of affordances, in particular when an attention mechanism focusing on objects is used. There are some previous studies linking affordance learning to intrinsic motivations and active learning (Ugur et al., 2007; Nguyen et al., 2013), but they did not investigate how intrinsic motivations can be used to learn object affordances when the visual sensors access only one object at a time. To face this condition, we will propose a mechanism that compares the estimated learning progress from acting on the currently seen object with the progress that it could gather by acting on other objects. The mechanism is inspired by the concept of *opportunity cost* used in economics, referring to the value of the opportunities that are lost by allocating a certain resource (here a unit of learning time) to a certain activity (Buchanan, 2008).

A second contribution of this work is the study of how the introduction of the attention mechanism, extracting information about the single object and about the object appearance/location impacts (a) the affordance learning process and (b) the second extrinsic phase where planning is needed to accomplish an extrinsic complex goal. The first issue has only been indirectly studied in the literature on affordances where models often assume pre-processing mechanisms to extract information on specific objects (see also the “OAC – Object Action Compound” framework pivoting on object information; Krüger et al., 2011). The second issue is important as attention is a key means to detect objects in humanoid robots (Camoriano et al., 2017) and information on objects is pivotal for both affordance detection (e.g., Montesano et al., 2008) and for planning. Regarding planning, attention can be used to refer to single objects, and then to reason about their relations, as typically done when using *structured representations* (Russell and Norvig, 2016). In particular, in the intrinsic phase the architecture presented here can use attention to identify different objects in the environment and learn affordances related to them. Then in the extrinsic phase it can use attention to parse the whole goal state into object-related sub-goals that can then be more easily accomplished one by one.

A third contribution of this work concerns the relationship between affordances and planning. In particular we will face the problem of what could be the utility of affordances, defined as the probability estimate of action success, within a planning system that is endowed with refined components implementing *forward models* and *relevance checking*. Are affordances still useful in such conditions? The importance of this problem derives from the fact that psychology shows that affordances are very important in real organisms. One might thus wonder if they can still furnish relevant functions within sophisticated systems endowed with the capacity of planning. In this respect, we will propose that: (a) affordances can play a role in forward planning as they support fast selection of relevant actions within the system's controller in a way similar to the way they are used to act in the environment, akin to the role of the “preconditions” of STRIPS-like operators in symbolic planning (Fikes and Nilsson, 1972); (b) affordances, when capturing the expected probability of action success, can play an important role in utility-based planning agents, the most sophisticated form of rational agent (Russell and Norvig, 2016).

The rest of the paper is organized as follows. Section 2 illustrates the experimental setup, and the architecture and functioning of the system. Section 3 shows the results of the tests. Section 4 compares the system proposed here to other systems proposed in the literature. Finally, section 5 draws the conclusions and illustrates open problems that might be tackled in the future.

## 2. Methods

### 2.1. Experimental Setup: Overview

The experimental scenario (Figure 1) consists of a black 2D working space containing different objects. Objects have different shapes (squares, circles, and rectangles) and colors (red, green, and blue). Section 2.2 describes the scenario and objects in more detail.

Each test consists of two phases: the *intrinsic phase* and the *extrinsic phase*. In the intrinsic phase the agent is free to interact with the objects for a certain time to autonomously acquire knowledge on them, in particular on their affordances and on forward models related to them (Figure 1A). In the extrinsic phase an overall goal state, with specific desired location and color of the objects in the working space, is presented to the agent that memorizes an image of it. The objects are then set in a different state and the agent has to re-create the goal state based on knowledge acquired in the first phase (Figure 1B).

The agent (section 2.3) is endowed with a simulated camera sensor that can look at different sub-portions of the working space, and is able to select and perform four actions on the object that is at the center of its camera. The actions can move the object to a new position or change its texture to a particular color (red/green/blue); different objects afford only a subset of these actions. As often assumed in the affordance literature, action execution is based on pre-programmed routines implementing the effect of the action that is selected and triggered.

Three versions of the system are implemented and compared: IGN, FIX and IMP. The three systems differ in the IM mechanisms they use to support affordance learning: FIX uses a mechanism taken from the literature (Ugur et al., 2007) whereas IGN and IMP use new mechanisms. Section 2.4 describes the three systems in detail. In the extrinsic phase, the knowledge acquired by the three systems in the intrinsic phase is tested with problems requiring goal-based or utility-based planning.

### 2.2. Working Space and Objects

The working space is formed by a 150 × 150 pixel square. The points of the working space are encoded as a 3D binary array where the first 2 dimensions encode the x-y pixel position, and the third dimension encodes the color (RGB). The color of the working space background is black. All objects are initially located on the vertexes of a 3 × 3 regular grid (white square in Figure 1).

Each object presents the following attributes: (a) center: x-y coordinates; (b) color: three values for red, green, and blue; (c) shape: either square, circle, or rectangle. Assuming the working space has a side measuring 1 unit, the circle has a diameter measuring 0.1 units, the square has a side measuring 0.1 units, and the rectangle has sides measuring 0.6 and 0.16 units.

As a consequence of the actions performed by the agent, the position and color of the objects can change. This defines the possible affordances of objects: “movable,” “greenable,” “redable,” and “bluable.” Each object has a specific subset of affordances, for example a blue circle is “movable” and “redable.” In some tests, affordances are stochastic in the sense that the related actions can produce an effect only with a certain probability.

### 2.3. The System Architecture

**Figure 3:**
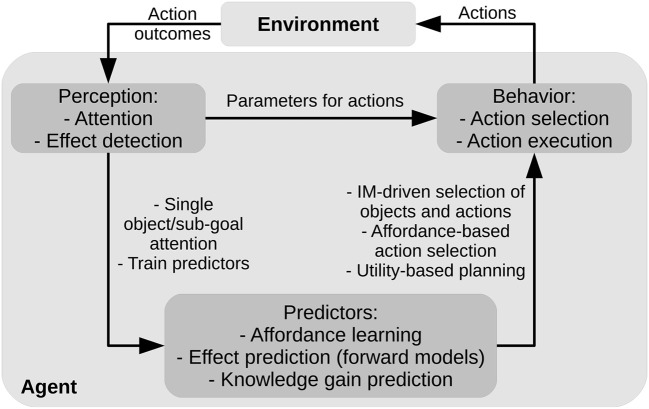
System architecture: main components.

The system controller consists of three different components (Figure 3): (a) the *perception component* implements a “bottom-up attention” mechanism that leads the system to scan the environment based on its salient features (color blobs of objects) and also observes the action effects in the environment by looking at portions of the scene that are changed by the actions (section 2.3.1); (b) the *action component* executes actions on the objects based on pre-wired routines (section 2.3.2); (c) the *predictor component* is formed by the predictors for affordances and forward models that support action selection in both the intrinsic and extrinsic phases (section 2.3.3). In the following sections we describe the different components in more detail.

#### 2.3.1. The Perception Component: Attention and Effect Detection

The perception component is responsible for the attention processes supporting visual exploration of both the environment and, in the extrinsic phase, the goal image. The perception component implements two attention processes: an inner attention process operating in parallel with an outer attention process. The outer attention scans the environment on the basis of two bottom-up processes both affecting gaze (they sum up): the first process is sensitive to the saliency of objects, and the second process is sensitive to the changes of the appearance of objects produced by actions. The inner attention scans the goal image on the basis of either the saliency of objects or by having the same focus as the one of the outer attention process. All these attentional processes are activated when needed on the basis of intrinsic motivations or planning processes, as we now illustrate more in detail.

Attention actively guides a RGB visual sensor (a pan-tilt camera) returning an image centered on the current attention focus and sufficient to always cover the whole working space independently of the gaze pointing. The central part of such an image, called *focus image*, forms the main input of the system. This focus image has a size of 0.14 × 0.14 (recall the scene is 1 × 1) and usually covers only one specific object. The whole image is used with a lower resolution (here in gray scale) to form a *peripheral image* that is used to drive the bottom-up attention processes now illustrated (cf. Ognibene and Baldassare, 2015).

The first type of bottom-up attention process, the saliency-based one, is driven by the most “salient” elements in the peripheral image, here the activation of pixels corresponding to objects. This process is implemented as follows. First a random noise (ϵ ∈ [−0.05, 0.05]) is added to each pixel of the peripheral image and the resulting image is smoothed with a Gaussian filter. Then the pixel with the maximum activation is used as the focus of attention (but if a change happens the second bottom-up attention mechanism also intervenes, see below). Thanks to the Gaussian smoothing, the focus falls around the center of the focused object, which thus becomes wholly covered by the focus image. The noise fosters exploration as it adds randomness to the saliency of objects, thus leading the system to explore the different objects.

Note that in the future more sophisticated approaches might be used to ensure that the focus-image involves only the focused object of interest (e.g., object-background segmentation approaches). Moreover, other mechanisms might be used to ensure a more efficient scan of the environment (e.g., inhibition of return might be used to avoid scanning the same location multiple times). These mechanisms are not considered here for simplicity and because the focus of this research is on the *effects* of attention, rather than on the mechanisms for its *control*.

The second bottom-up attention process, sensitive to changes, is directed to detect the effects of actions. The process works as follows. Firstly the system focuses on the portion of space where a *change* in the periphery image takes place (this mimics some processes of primates for which a reflex focuses attention on changes happening in the environment (Comoli et al., 2003; Gandhi and Katnani, 2011; Sperati and Baldassarre, 2014). To this purpose, the system computes the “change image” given by the pixel-by-pixel absolute difference between the whole periphery image after and before the performance of the action: both images are taken with the same initial gaze before the next attentional movement but the “after-image” is taken after the action performance (e.g., leading to displace the object). The change image is smoothed with a Gaussian filter and summed up to the salience-based attention image described above to guide the gaze displacement to the maximally activated pixel of the “sum image.” This process leads the system to look at the object that has been changed by the action, e.g., the object that has been changed in color or displaced in space (in the latter case, the system gazes the position where the object is moved, and not the position where it disappears, due to the object saliency that is not present where it disappears). After this attentional movement on the changed object is performed, the system compares the focus image (involving only the area covering the object) and the object position (given by the gaze direction) before and after performing the attentional movement itself: this allows the system to decide if the performed action was successful or not (presence of affordance). The focus image comparison is based on the L1 norm of the difference between the vectors of the two images before and after action performance, divided by the number of dimensions of the vectors: if this measure is higher than τ_i_ = 10^−5^ then the focus image is considered to have changed. The position comparison is based on the L1 norm of the difference between the vectors of the two (x,y) focus positions before and after action performance, divided by 2: if this measure is higher than τ_*p*_ = 0.02 then the position of the object is considered to have changed.

An affordance is considered to be in place if: (a) an effect is detected; (b) the effect is the one related to the performed action (e.g., the object is displaced by the action “move,” or the object is made green by the action “change object color to green”). This information is used to train the affordance predictors. The focus image and object position after the action execution are used to train the effect predictors.

#### 2.3.2. The Action Component

The system is equipped with four actions: move object, change object color to green, change object color to red, and change object color to blue. The move action can displace objects in the environment if they have the affordance for this effect. The move action is parametric: it affects the target object (object under focus) on the basis of two parameters corresponding to the object desired x-y location. During the intrinsic phase, the desired location is randomly generated within the working space (excluding positions that cause object overlapping). During the extrinsic phase, the target location corresponds to the location of the “sub-goal” that the system is currently attempting to accomplish (see section 2.4.3). The color-changing actions are non-parametric: they simply change the color of the target object into the desired one if the object has the affordance for the corresponding effect. Only one color-change action might have been considered if parameterized with the color (this would have been a discrete parameter vs. the continuous parameters of the move action). We chose a non-parametric version of the color actions to develop the features of the system working for both parametric and non-parametric actions.

#### 2.3.3. The Predictor Component: Forward Models and Affordances

The predictor component is formed by 16 predictors (these are regressors), 4 for each of the 4 actions: (a) *the affordance predictor* predicts the object affordance (i.e., the probability that the action effect takes place when the action is performed); (b) *the learning-progress predictor* predicts the learning progress of the affordance predictor when applying the action to the target object, and is used to generate intrinsic motivations based on the learning progress of the affordance predictors; (c) *the what-effect predictor* predicts the focus image of the object resulting after the action performance; (d) *the where-effect predictor* predicts the object position resulting after the action performance. Given the simplicity of the stimuli, the predictors are implemented here as simple perceptrons but more sophisticated models might be used to face more challenging scenarios. All the predictors are trained during the intrinsic exploration phase and are now explained more in detail.

The affordance predictors estimate the affordance probability $Pr\left(s_{b,o}^{\prime }\in G|a,s_{b,o}\right)$ of each action/goal related to different objects. Each predictor gets as input the focus image (whose pixels are each mapped onto (0, 1) and unrolled into a vector) and returns, with one output sigmoid unit, the prediction of the action success. Each predictor is trained with a standard rule and a learning target 0 or 1, encoding respectively the failure or success of the action to produce its desired effect, i.e., the presence/absence of the affordance (the learning rate used varied in the different tests, see section 3).

Each learning-progress predictor gets as input the focus image and returns, with a continuous linear output unit, the learning progress of the associated affordance predictor. The predictor is updated with where the target for learning is the difference in the output of the corresponding affordance predictor, computed before and after the action is performed and before the affordance predictor is updated.

Each of the what-effect predictors gets as input the focus image and predicts, with sigmoidal output units, the focus image after the action performance. The predictor is updated with rule with a target corresponding to the observed focus image after the action is performed.

Each of the where-effect predictors gets as input the initial (x, y) position of the target object and the desired (x, y) position of the object depending on the sub-goal, and predicts, with two linear units, the predicted object (x, y) position after the action performance [x and y coordinates are each mapped to the range (0, 1)]. The predictor is updated with a where the target for learning is the object position after the action is performed.

### 2.4. The Intrinsic-Phase Learning Processes and the Extrinsic-Phase Planning

In this section we first present the motivation signals (section 2.4.1) and the algorithm for learning affordances and forward models (section 2.4.2) used by the three versions of the system (IGN, FIX, and IMP) during the intrinsic phase. Then we describe the two algorithms of the attention-based goal planner (section 2.4.3) and the attention-based utility planner (section 2.4.4) used in the extrinsic phase.

#### 2.4.1. IM Signals

In the intrinsic phase, the system autonomously explores the objects in the environment to learn affordances and train its predictors. The exploration process is driven by IMs related to the knowledge acquired by the affordance and learning-progress predictors. Depending on how the IMs are implemented, we have three versions of the system: FIX, IGN, and IMP.

The FIX system uses an IM mechanism for affordance learning like the one used by Ugur et al. (2007). This work studies a mobile robot that learns the “traversability” affordance in a maze scattered with obstacle-objects. A Support Vector Machine (SVM) is used to classify the view of the obstacle-objects to estimate the presence/absence of the affordance. The system estimates the novelty, and hence the interest, of the current view of the objects on the basis of its distance from the hyperplane used by the SVM to classify the affordance presence/absence. If this distance is below a fixed threshold, the view is considered interesting and so the system performs the exploratory action of trying to traverse the maze. The system observes if the affordance holds, and uses its observation to train the SVM.

In our case, as a measure of how interesting the current object is we consider the Shannon entropy of the estimated affordance probability:

(1) $$\begin{array}{l} H\left(p\right)=-\overset{n}{\underset{i=1}{\sum }}Pr\left(x_{i}\right)log_{b}\left(Pr\left(x_{i}\right)\right)=-p\log_{2}p-\left(1-p\right)\log_{2}\left(1-p\right) \end{array}$$

where we considered *b* = 2 as the basis of the logarithm; *x*_*i*_ are the two events $s_{b,o}^{\prime }\in G$ and $s_{b,o}^{\prime }\notin G$ corresponding to the presence or absence of the affordance, having respectively a probability $Pr\left(s_{b,o}^{\prime }\in G\right)=p$ and $Pr\left(s_{b,o}^{\prime }\notin G\right)=1-p$. The use of this formula is justified by the fact that entropy is a measure of ignorance (uncertainty) of the system: the uncertainty is minimal when *p* = 0 or *p* = 1, and maximal when *p* = 0.5 (the value of the entropy is here normalized so that *H*(*p*) ∈ (0, 1), in particular *H*(0) = *H*(1) = 0 and *H*(0.5) = 1). The weights of the affordance predictors are initialized to 0, resulting in an initial sigmoid activation of 0.5 and an ignorance of 1.

Following Ugur et al. (2007), the current object is considered interesting, and hence worth exploring, when the entropy is above a threshold *th* (here *th* = 0.3, which corresponds to an ignorance value, i.e., affordance predictor output, of 0.947 or 0.053). Ignorance (entropy) thus represents the IM signals that drive exploration of objects. Since we have more than one action, for a given focused object we consider the action with maximum ignorance for that object. This mechanism is simple and interesting, but it also has some limitations when applied to multiple objects and actions as it leads to an evaluation of how interesting potential experiences are in a fixed way. In particular, all objects with an ignorance above the threshold will be considered equally interesting. Moreover, after the ignorance related to an object decreases below the threshold the agent will stop exploring it independently of the fact that it might still have some exploration time available.

The IGN system (IGN stands for “IGNorance”) is a first version of our system that is directed to overcome the limitations of the IM mechanism of FIX. The new mechanism uses a dynamic threshold *th*. This threshold is continuously adjusted as a leaky average of the IM signal, *IM* (here *IM* = *H*), related to the objects explored one after the other in time:

(2) $$\begin{array}{l} th_{t}=th_{t-1}+\nu \left(-th_{t-1}+IM_{t-1}\right) \end{array}$$

where *t* is a trial and ν the leak coefficient (ν = 0.3 in the deterministic environment and ν = 0.1 in the stochastic environment). When objects are explored and predictors are trained, the ignorance of objects, and hence the IM signal related to them, will decrease and so will *th*. By comparing the highest action-related entropy of an object with *th* the system will be able to trigger the exploration action or to pass to explore more interesting objects. When the system decides to not explore the object, then the leaky average gets *IM*_*t*−1_ = 0 as input. This implies that when objects are considered not interesting, the threshold progressively decreases so that some objects become interesting again.

A limitation of the IM mechanisms of IGN is that it is not able to cope with stochastic environments where the success of an action is uncertain (e.g., when the agent tries to move an object, the object moves only with a certain probability). In this case, the affordance predictor will tend to converge toward the corresponding probability *p* and so objects with stochastic affordances will always remain interesting.

The IMP system (IMP stands for “IMProvement”) overcomes the limitations of IGN by suitably coping with stochastic environments. The motivation signal used by IMP is implemented as the absolute value of the learning-progress predictor output (let's call this *LP*). Similarly to IGN, *th* is computed as a leaky average of the IM signal, namely with Equation (2) where *IM* = *LP* (ν = 0.1 in all tests). In particular, as for IGN the expected learning progress of the action with the highest *LP* is compared with *th*. This makes the system explore the current object if it promises a learning progress that is higher than the learning progress for other objects, represented by *th*. The learning-progress predictor weights are initialized to random values within (−0.00075, +0.00075) so that the motivation signal is non-zero for all objects when the intrinsic phase starts. The distinction between IGN and IMP reflects the distinction between *prediction error* and *prediction error improvement* within the literature on IMs, justified as here by the need to face deterministic or stochastic environments (Schmidhuber, 1991a; Santucci et al., 2013).

The mechanism of the leaky average threshold, used in IGN and IMP, allows the agent to indirectly compare the relative levels of how interesting different objects are, and to focus the exploration effort on the most interesting of them notwithstanding the fact that *different objects are in focus at different times* due to the presence of the active vision mechanisms. This is a novel general mechanism that allows the integration of attention (targeting different objects) and IMs (returning an interest of the object). Here the mechanism is used for the learning of object affordances but its generality allows its use also for the acquisition of other types of knowledge in the presence of focused attention.

#### 2.4.2. Intrinsic Phase: Learning of Affordances and World Model

The intrinsic phase allows the system to autonomously explore the objects by looking at and acting upon them. Based on the observed consequences of actions, this allows the system to train the predictors, including those related to affordances. At each step of the phase, the system performs a number of operations as illustrated in Algorithm 1. This algorithm is used by both the goal-based planner and the utility-based planner.

**Algorithm 1:** Intrinsic phase: one step of learning of affordances and forward models

1:	(object_image, object_position) ← Scan(environment)	
2:	(action, motivation_signal) ← SelectActionWithHighestIM(action_list, predictors, object_image, object_position)	
3:	**if** (motivation_signal ≥ motivation_threshold) **then**	
4:	ExecuteAction(action, object_image, object_position)	
5:	(new_object_image, new_object_position) ← ScanEffect(new_environment, environment)	
6:	affordance ←…	
7:	Affordance(action, new_object_image, new_object_position, object_image, object_position)	
8:	UpdateWeights(affordance_predictor, action, object_image, affordance)	
9:	UpdateWeights(affordance_predictor, action, object_image, affordance, improve_predictor)	⊳ Only IMP
10:	**if** (affordance = TRUE) **then**	
11:	UpdateWeights(effect_predictors, action, object_image, object_position,…	
12:	new_object_image, new_object_position)	
13:	**end if**	
14:	motivation_threshold ← LeakyAverage(motivation_threshold, motivation_signal)	⊳ Only IGN/IMP
15:	**else**	
16:	motivation_threshold ← LeakyAverage(motivation_threshold, 0)	⊳ Only IGN/IMP
17:	**end if**	

The algorithm is based on the following operations and functions: (a) *the Scan* function focuses the system visual sensor on an object based on the bottom-up attention mechanism and returns the image and position of the object; (b) *SelecActionWithHighestIM* selects the action with the highest IM and computes its motivation signal; (c) *ExecuteAction* executes the selected action as illustrated in section 2.3.2 if the motivation signal is higher than the threshold; (d) *ScanEffect* looks where a change in the environment has happened (e.g., at the new position occupied by the object after the move action, or at the object that changed its color after a change-color action), and returns the resulting new object image and position; (e) *Affordance* compares the past and new state of the target object and returns a Boolean value (affordance) for the affordance presence/absence; (f) *UpdateWeights* updates the connection weights of the predictors of the performed action as illustrated in section 2.3.3: the affordance predictor is updated on the basis of the affordance presence/absence; in the case of IMP, the learning-progress predictor is also updated on the basis of the affordance predictor output before and after the action performance (and hence the predictor update); in the case of the presence of the affordance (action success), the effect predictor learns to predict the effects of the performed action; (g) *LeakyAverage*, present in the case of IGN/IMP, updates the threshold according to Equation (2) and uses as input either the IM signal of the performed action, or zero if no action was executed.

#### 2.4.3. Extrinsic Phase, Pursuing the Overall Goal: Goal-Based Planner

During the extrinsic phase, the system is tested for its capacity to accomplish an “overall goal” based on the knowledge acquired during the intrinsic phase. Such an overall goal is assigned to the agent through the presentation of a certain desirable spatial/color configuration of some objects in the environment. The agent stores the goal as an image (“goal image”). The configuration of the objects is then changed and the task of the agent is to act on the environment to arrange it according to the goal image.

Importantly, the agent scans the goal image through a second “inner” attention mechanism similar to the “outer” attention mechanism used to scan the external environment. This inner attention mechanism is important to parse the goal image into *sub-goals*, each corresponding to the configuration of a single object in the goal image, one per saccade. These sub-goals are then pursued in sequence to accomplish the overall goal.

The operations taking place in one step of this process are shown in detail in Algorithm 2. The pseudo-code of the algorithm highlights the effects of the factorization of information on objects, related to their state and position, based on the attention mechanisms. A new sub-goal is selected either in the case that the previous sub-goal has been accomplished (in which case the Boolean variable *sub*_*goal*_*active* = *FALSE*) or if a time out elapses in the unsuccessful attempt to pursue it (here the time out is equal to 8 iterations of the algorithm). The agent uses the function *Scan* to identify a new target sub-goal: this function scans the goal-image with the saliency-based attention mechanism and returns the new sub-goal image and focus location (*sub*_*goal*_*image* and *sub*_*goal*_*position*).

**Algorithm 2:** Extrinsic phase: one step of goal-based planning

**if** time_out OR (NOT sub_goal_active) **then**	⊳ Select non-achieved sub-goal
(sub_goal_image, sub_goal_position) ← Scan(goal)	
focus_image ← ScanEnvironmentWithSameFocusAsSubGoal(environment)	
sub_goal_active ← GoalNotAchievedCheck(sub_goal_image, focus_image)	
**end if**	
**if** (sub_goal_active = TRUE) **then**	
(object_image, object_position) ← Scan(environment)	⊳ Select object
(sub_goal_achievable, action) ← ActionPlanning(predictors, action_list,…	
(sub_goal_image, sub_goal_position, object_image, object_position)	⊳ Plan action
**if** (sub_goal_achievable = TRUE) **then**	
ExecuteAction(action, object_image, object_position, action)	⊳ Perform action
focus_image ← ScanEnvironmentWithSameFocusAsSubGoal(environment)	
sub_goal_active ← GoalNotAchievedCheck(sub_goal_image, focus_image)	
**end if**	
**end if**	

Next, the agent checks if the sub-goal has not been accomplished yet. To this purpose, the function *ScanEnvironmentWithSameFocusAsSubGoal* drives the outer attention focus (targeting the environment) to the position corresponding to the inner attention focus (targeting the goal image) and returns the corresponding focus image (*focus*_*image*). Then the function *GoalNotAchievedCheck* compares the *sub*_*goal*_*image* and the *focus*_*image* to check that the sub-goal has not been achieved yet, in particular it sets the variable *sub*_*goal*_*active* to *FALSE* or *TRUE* if they, respectively, match or mismatch (the match holds if the Euclidean distance between the vectors corresponding to the two images is below a threshold τ = 0.01).

If the sub-goal has not yet been accomplished, the system scans the environment to find a new object and then the function *ActionPlanning* checks if at least one action is able to accomplish the sub-goal by acting on the focused object. To this purpose, the function uses the effect predictors to predict the effect of each action and then compares it with the sub-goal (this happens if the Euclidean distances are below 0.0035 for the sub-goal image and the object image, and below 0.01 for their position coordinates). Depending on the result of the planning, the function sets the Boolean variable *sub*_*goal*_*achievable* to *TRUE* or *FALSE* and possibly returns the action to be executed to achieve the sub-goal. These processes, based on the forward models, are an important part of the algorithm as they implement a one-step forward planning process.

Lastly, if a potentially successful action has been identified, it is executed and then the system checks again if the sub-goal has been accomplished. If so, *sub*_*goal*_*active* is set to *FALSE* so that a new sub-goal is chosen in the next iteration.

Importantly, the *ActionPlanning* function could possibly check all actions. Affordances (i.e., the predictors estimating *Pr*(*g*_*j*_|*a*_*j*_, *o*_*i*_)) can be employed to avoid this. In particular, the check can be limited only to those actions having an affordance for the current object (here when *Pr*(*g*_*j*_|*a*_*j*_, *o*_*i*_) > 0.5). This allows affordances, which are computed fast through 1-output neural networks, to speed up the planning search by reducing the number of more computationally expensive operations involving the prediction of action effects (new object image and position) and their comparison with the sub-goal. Here actions are only 4, but this advantage increases with the number of actions available to the agent.

#### 2.4.4. Extrinsic Phase, Pursuing the Overall Goal: Utility-Based Planner

In the case of *utility planning*, different “sub-goals” deliver a different value if accomplished. In cases where the agent has a limited amount of resources available to accomplish the goals (e.g., time or energy to perform actions), it should first invest such resources in the accomplishment of the most valuable sub-goals (for simplicity, we assume here a constant cost per action and a negligible cost of reasoning with respect to acting, as often done in utility-based planning, Russell and Norvig, 2016). Notice that in utility planning “sub-goals” are directed to accomplish the overall objective of utility maximization rather than an overall goal intended as a particular state of the environment.

The utility-based planner works as shown in Algorithm 3. When the Boolean variable *max*_*utility*_*estimatation* is *TRUE*, the planner evaluates the value of the possible sub-goals it can achieve with the available object-action combinations and stores an estimate of its value in the variable *potential*_*utility*, otherwise it acts in the world. Various mechanisms could be used to set and keep the system in the evaluation mode: here for simplicity we gave the system a certain amount of iterations before performing an action, but more flexible mechanisms might be used (e.g., passing to act when the estimates stabilize). To perform this evaluation, the system performs the sub-goal seeking, object seeking, and one-action planning processes as done in Algorithm 2. However, instead of executing the planned actions the system only updates the *potential*_*utility* if the current goal-object couple has a higher utility than it: this ensures that the potential utility estimation tends to approximate the value of the most valuable sub-goals. The utility of the sub-goal-object couple, given the found action, is computed as:

(3) $$\begin{array}{l} U=Pr\left(s_{b,o}^{\prime }\in G|a,s_{b,o}\right)\times V\left(s_{b,o}^{\prime }\right) \end{array}$$

where $Pr\left(s_{b,o}^{\prime }\in G|a,s_{b,o}\right)$ is the affordance (expected probability of accomplishing the desired sub-goal) and $V\left(s_{b,o}^{\prime }\right)$ is the value of the sub-goal. The actual update of the potential utility is based on a leaky average (based on Equation 2 using a leak rate ν = 1.0; a value lower than this might be used for having a more reliable but slower process).

**Algorithm 3:** Extrinsic phase: one step of utility-based planning

**if** (time_out OR (NOT sub_goal_active)) **then**	⊳ Select non-achieved sub-goal
(sub_goal_image, sub_goal_position) ← Scan(goal)	
focus_image ← ScanEnvironmentWithSameFocusAsSubGoal(environment)	
sub_goal_active ← GoalNotAchievedCheck(sub_goal_image, focus_image)	
**end if**	
**if** (sub_goal_active = TRUE) **then**	
(object_image, object_position) ← Scan(environment)	⊳ Select object
(sub_goal_achievable, action) ← ActionPlanning(predictors, action_list,…	
sub_goal_image, sub_goal_position, object_image, object_position)	⊳ Plan action
**if** (sub_goal_achievable = TRUE) **then**	
object_utility ← ComputeUtility(object_affordance, sub_goal_value)	
**if** (max_utility_estimatation = TRUE) **then**	⊳ Computing the maximum possible utility
**if** (object_utility ≥ potential_utility) **then**	
potential_utility ← LeakyAverage(potential_utility, object_utility)	⊳ Increase utility expectation
**end if**	
**else**	⊳ Acting if high utility is attainable
**if** (object_utility ≥ potential_utility) **then**	
ExecuteAction(object_image, object_position, action)	
**end if**	
potential_utility ← LeakyAverage(potential_utility, 0)	⊳ Decrease utility expectation
**end if**	
sub_goal_active = FALSE	
**end if**	
**end if**	

When the *max*_*utility*_*estimation* is set to *FALSE*, the system starts to perform actions in the environment. This is similar to what is done in Algorithm 2, the difference being that now the system executes actions only if the expected utility of the current subgoal-object is higher than the potential utility. For each chosen action, this potential utility is decreased with the leaky average using 0 as input and a leak rate ν = 0.1. This ensures that the potential utility progressively decreases so that the system initially works on most valuable sub-goals-object couples and then engages with less valuable ones.

## 3. Results

To test the performance of the systems, different tests were run with both deterministic and stochastic environments. Performance in the intrinsic phase was measured by evaluating the quality of the output of the predictors when receiving as input each one of the nine focused images corresponding to the nine possible objects (Figure 1).

### 3.1. Deterministic Environment

**Figure 4:**
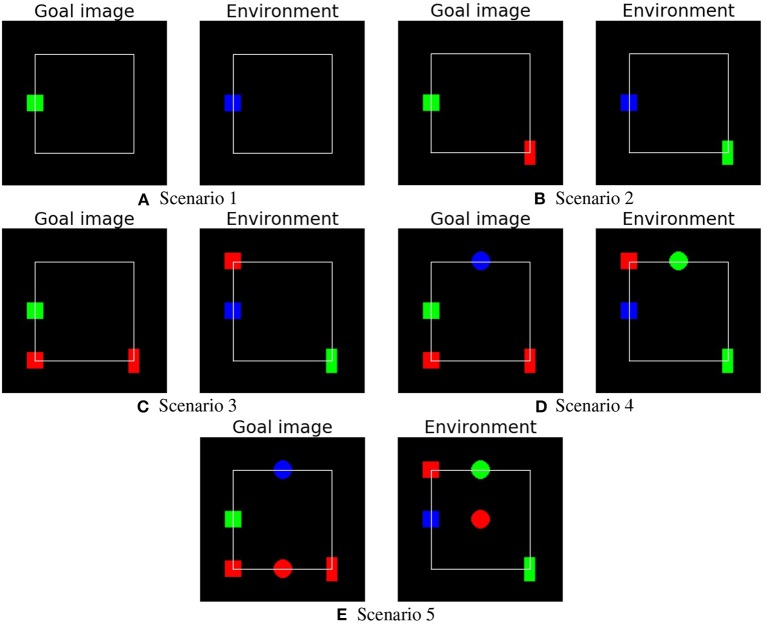
The five different scenarios (goal image and initial environment setup) used to test the systems in the extrinsic phase. The scenarios involve an increasing number of objects. **(A)** Scenario 1. **(B)** Scenario 2. **(C)** Scenario 3. **(D)** Scenario 4. **(E)** Scenario 5.

In the deterministic environment two tests were run to test the goal-based planner. The first, called the *base test*, involved all nine objects each affording all four actions. The purpose of this test is to compare the different intrinsic motivation mechanisms driving affordance acquisition. During the intrinsic phase of this test, the objects are initialized as in Figure 1A and the system explores them. Afterwards, in the extrinsic phase the system is tested in five different conditions involving different goal images and environment settings (Figure 4). The first scenario contains only one object, a blue square that has to be changed to green. In each subsequent scenario one additional object is introduced to increase the scenario difficulty: scenario 2 introduces a green rectangle that has to be changed to red; scenario 3 introduces a red square that has to be moved; scenario 4 introduces a green circle that has to be changed to blue; finally, scenario 5 introduces a red circle that has to be moved.

The second test, called the *late object test*, involves an intrinsic phase where some objects, not initially present, are introduced after the system has acquired knowledge on objects introduced initially. Then the system is tested with the extrinsic phase scenarios as in the base test (Figure 4). The purpose of this test is to evaluate how the systems perform when, during the intrinsic phase, new knowledge is added to already acquired knowledge, a situation very common in open-ended learning conditions.

Affordance predictor learning rates were α = 0.01 in the IGN and IMP systems and α = 0.002 in the FIX system. The learning rates of the learning-progress predictors were set to α = 0.005. The leaky average of the intrinsic motivation was updated with a leak rate ν = 0.3 in the IGN system and ν = 0.1 in the IMP system. The results of these tests are presented in the following sections.

#### 3.1.1. Base Test

In the base test, all the nine objects afford all the four actions with the exception of those not causing any change (Table 1).

**Table 1:** Base test: affordance probabilities for all objects and actions.

**Object**		**Move action prob**.	**Turn green prob**.	**Turn red prob**.	**Turn blue prob**.
1	Red square	1.0	1.0	0.0	1.0
2	Green square	1.0	0.0	1.0	1.0
3	Blue square	1.0	1.0	1.0	0.0
4	Red circle	1.0	1.0	0.0	1.0
5	Green circle	1.0	0.0	1.0	1.0
6	Blue circle	1.0	1.0	1.0	0.0
7	Red rectangle	1.0	1.0	0.0	1.0
8	Green rectangle	1.0	0.0	1.0	1.0
9	Blue rectangle	1.0	1.0	1.0	0.0

**Figure 5:**
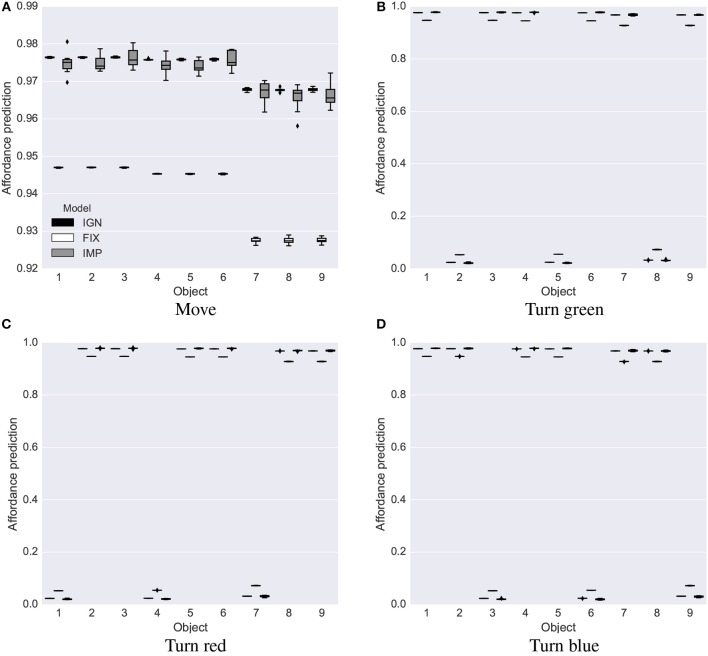
Base test: affordance predictions (y-axis) after 6,000 learning steps for the four actions (four graphs) and nine objects (x-axis) averaged over 10 trials in the base test, for the IGN, FIX, and IMP systems. Mid-line of boxes shows median values, boxes show quartiles, and bars show the min-max range. The target values that the predictors had to estimate were 0 or 1. **(A)** Move. **(B)** Turn green. **(C)** Turn red. **(D)** Turn blue.

**Intrinsic phase**. The intrinsic phase of the base test was run 10 times, each lasting 6,000 steps, for each system, IGN, FIX and IMP. All three systems learned a good estimate of the affordances of the nine objects (Figure 5; the true values to estimate are either 0 or 1). The affordance predictions after learning were closer to the true values for the IGN and IMP systems compared to the FIX system. The variance of the predictions was lower in the FIX and IGN systems compared to the IMP system Figure 5A.

These results offer a first validation of the idea that the IMP and ING systems, using a dynamic threshold for evaluating the interest of the current object in terms of its potential return of information, outperform the FIX system previously proposed in the literature. The reason is that the IMP and IGN systems can decide to explore or ignore an object based on the possibility of learning more from other objects, rather than in absolute terms as in FIX.

**Extrinsic phase**. The test of the extrinsic phase was repeated 10 times for each system, for each extrinsic scenario, and for each of the 10 repetitions of the intrinsic phase. The results show that the three systems succeeded in accomplishing the tasks in the majority of times (Table 2).

**Table 2:** Base test: success of the extrinsic-learning process for the three systems IGN, FIX, and IMP.

	**Extrinsic-phase scenarios**				
**System**	**1**	**2**	**3**	**4**	**5**
IGN	1.0	1.0	1.0	1.0	0.9
FIX	1.0	1.0	1.0	1.0	0.8
IMP	1.0	1.0	1.0	1.0	0.9

*For each of the 10 intrinsic-phase runs and each of the 5 extrinsic-phase scenarios, the extrinsic-phase test was repeated 10 times: the figures reported here represent the portion of “successful” intrinsic-phase runs leading to > 90% successful extrinsic-phase tests*.

**Figure 6:**
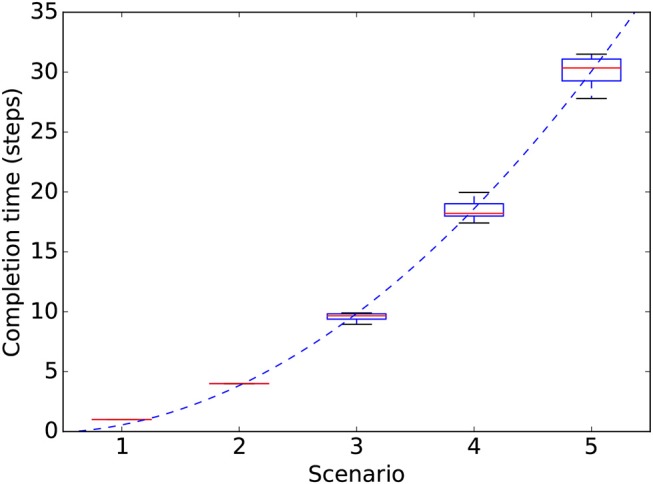
Completion times (y-axis) for the IMP system in the different extrinsic-phase scenarios involving an increasing number of objects (x-axis). Data refers to 100 simulations (10 runs of the extrinsic-phase test for each of the 10 runs of the intrinsic-phase learning process). For each scenario, the mid-line of boxes shows median values, boxes show quartiles, and bars show the min-max range. The dashed line shows a quadratic fit: *y* = *ax*^2^ + *bx* = 1.364*x*^2^ − 0.808*x*.

Completion time in the IMP system showed an approximately quadratic dependency on the number of sub-goals (Figure 6) and also an increasing variance. The other two systems showed similar results. This test suggests that in a more complex environment with more objects, the system would be incapable of completing tasks within a reasonable amount of time. The reason for the poor scaling is mainly due to the simple bottom-up attention mechanism used here to guide attention, which uses a random exploration to find the objects needed to accomplish a certain sub-goal. A top-down mechanism capable of avoiding multiple explorations of the same objects would supposedly lead to a linear dependence of the completion time on the number of sub-goals.

#### 3.1.2. Late-Object Tests

Three late-object tests were run. The features of the tests are summarized in Table 3. In the first test the six red-green-blue square and circle objects are present from the start of the simulation and have all affordances, while the red-green-blue rectangle objects are introduced late, have move affordance set to 0 (not movable) and the color affordances set to 1 (“greenable,” “redable,” and “blueable”). In the second test, the square objects are present from the start and afford all actions, the circle objects are present from the start and do not afford any action, and the three rectangle objects are introduced late and afford all actions. In the third test, the square and rectangle objects are present from the start and afford all actions, while the three circle objects are introduced late and do not afford any action. The three tests were run for 10,000 steps each and late objects were introduced after 2000 steps.

**Table 3:** The structure of the three late-object tests.

	**Objects types:**		
**Late-test number**	**Squares**	**Circles**	**Rectangles**
	Start	Start	Late
1	Move: 1	Move: 1	Move: 0
	Color: 1	Color: 1	Color: 1
	Start	Start	Late
2	Move: 1	Move: 0	Move: 1
	Color: 1	Color: 0	Color: 1
	Start	Late	Start
3	Move: 1	Move: 0	Move: 1
	Color: 1	Color: 0	Color: 1

“*Start/Late”: objects that are present form the start of training/introduced later. “Move/Color”: type of affordances. “1/0”: presence/absence of the affordances*.

**Figure 7:**
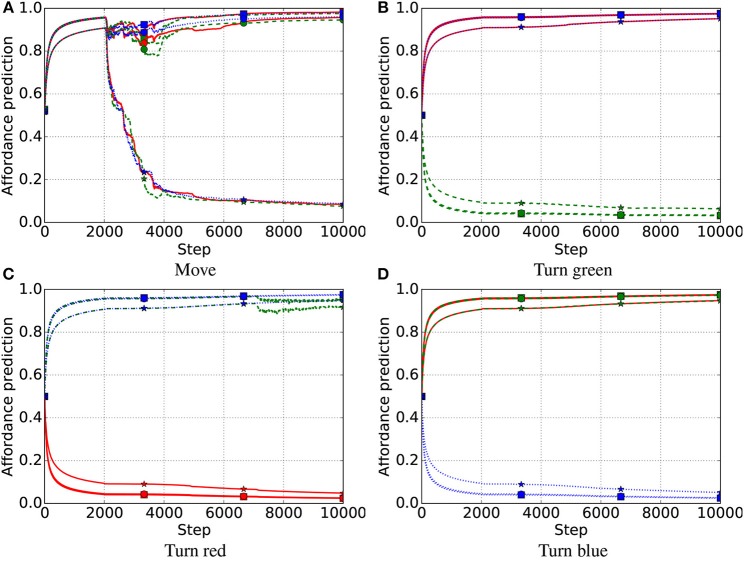
First late-object test, IGN system. Affordance prediction for the four actions (4 graphs) and nine objects (lines in each graph) averaged over 10 trials. Red lines refer to red objects, green dashed lines to green objects, and blue dotted lines to blue objects. Markers on lines represent the shape of objects, where squares refer to square objects, circles to circular objects, and stars to rectangular objects. Note that an object of a color does not have the affordance to be turned to the same color (e.g., a red object cannot be turned red) as this involves no change. **(A)** Move. **(B)** Turn green. **(C)** Turn red. **(D)** Turn blue.

**Figure 8:**
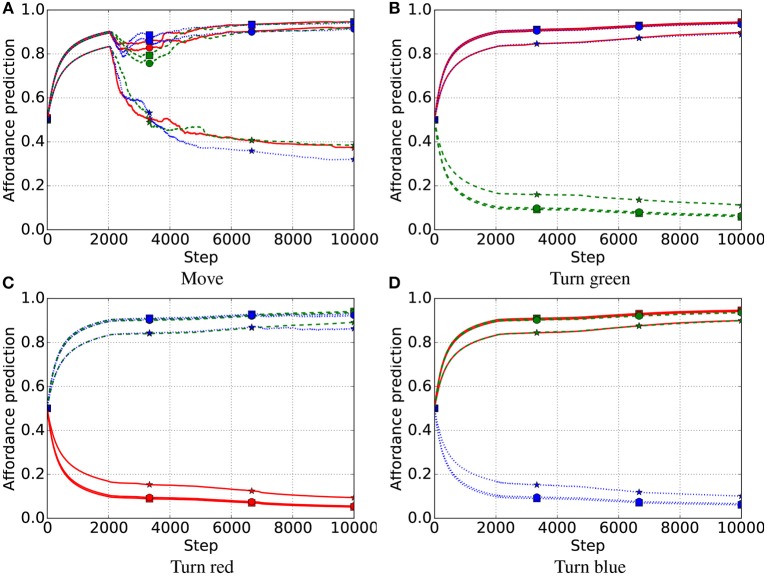
First late-object test, FIX system. Red lines refer to red objects, green dashed lines to green objects, and blue dotted lines to blue objects; markers on lines represent the shape of objects, where squares refers to square objects, circles to circular objects, and stars to rectangular objects. **(A)** Move. **(B)** Turn green. **(C)** Turn red. **(D)** Turn blue.

**Figure 9:**
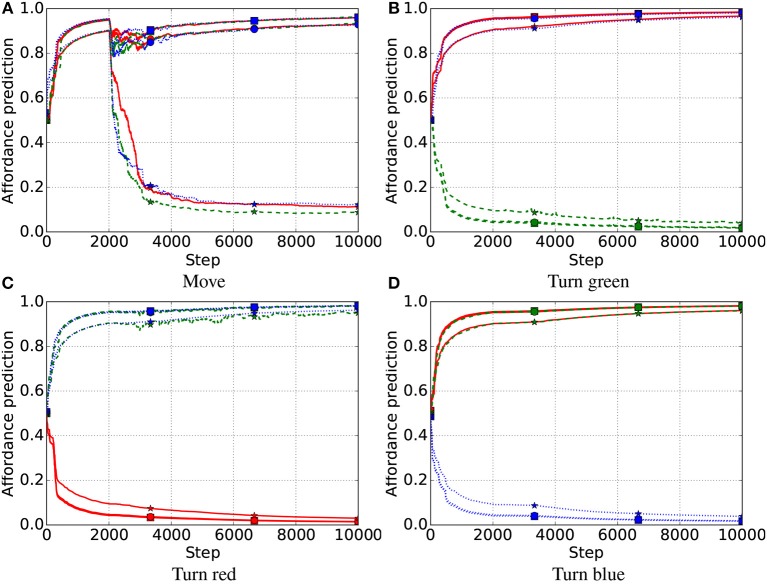
First late-object test, IMP system. Affordance prediction for the four actions (4 graphs) and nine objects (lines in each graph) averaged over 10 simulations. Red lines refer to red objects, green dashed lines to green objects, and blue dotted lines to blue objects; markers on lines represent the shape of objects, where squares refers to square objects, circles to circular objects, and stars to rectangular objects. **(A)** Move. **(B)** Turn green. **(C)** Turn red. **(D)** Turn blue.

**Figure 10:**
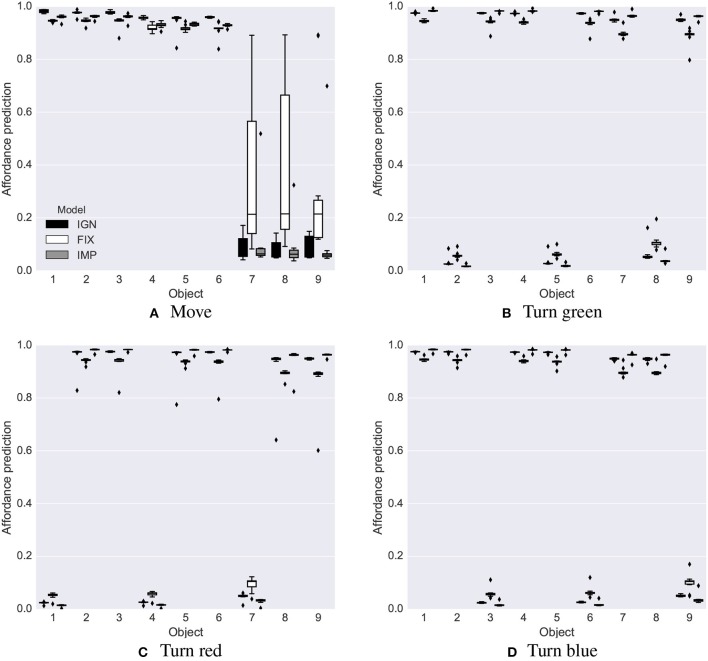
First late-object test: affordance predictions after learning. Plotted as in Figure 5. **(A)** Move. **(B)** Turn green. **(C)** Turn red. **(D)** Turn blue.

In the first late-object test, all three systems successfully learned the affordances of all objects, including the non-movable rectangles introduced late (Figures 7–9). After the intrinsic phase, the predictions of object affordances were correct for all three systems, except the “move” action affordances of the late objects 7 and 8 in the FIX system, which also showed the highest variance, followed by the IMP system (Figure 10).

Performance in the five extrinsic phase test scenarios (Table 4) was low for the FIX system compared to the IGN and IMP systems, and was highest for IMP.

**Table 4:** First late-object test: success of the three systems IGN, FIX, and IMP in the five extrinsic scenarios.

	**Extrinsic-phase scenario**				
**System**	**1**	**2**	**3**	**4**	**5**
IGN	1.0	0.4	0.4	0.4	0.1
FIX	0.8	0.0	0.0	0.0	0.0
IMP	1.0	0.9	0.9	0.9	0.6

*Data as in Table 2*.

In the second and third late-object test, the three systems differed in their behaviors while learning the affordances during the intrinsic phase, but all presented a similar performance when tested in the extrinsic phase, so we report the data related to them in Supplementary Material. In the second late-object test, the IGN and IMP systems first learned the affordances of objects introduced early in the simulations, and then focused and learned the affordances of the objects introduced late (Supplementary Figures 1, 2). Instead, the FIX system did not have such efficient focus (Supplementary Figure 3). After learning, the affordance predictions were correct for the IGN and IMP systems (with a higher variance for the IMP system) whereas the FIX system was less accurate and had a higher variance (Supplementary Figure 4).

Regarding the extrinsic-phase tests (Table 5), all three systems were successful in the first three scenarios, but failed in the fourth and fifth scenarios, showing that the different quality of affordances acquired in the intrinsic phase did not affect the performance in these particular tests. All systems failed the extrinsic phase scenario 4 and 5, involving an additional circle each, because in the late-object tests 2 and 3 the circle objects do not afford any action and so their state cannot be changed.

**Table 5:** Second late-object test: success of the three systems IGN, FIX, and IMP in the five extrinsic scenarios.

	**Extrinsic-phase scenario**				
**System**	**1**	**2**	**3**	**4**	**5**
IGN	1.0	1.0	1.0	0.0	0.0
FIX	0.9	0.9	0.7	0.0	0.0
IMP	1.0	0.9	0.9	0.0	0.0

*Data as in Table 2*.

The first and second late-object tests confirm that the IGN and IMP systems outperform the FIX system in learning affordances as they can decide to explore a certain object on the basis of a comparison between its expected information gain and the information gain expected on average from other objects.

In the third late-object test, none of the systems successfully learned to focus on, and predict accurately, the lack of affordances of the late circle objects (Supplementary Figures 5–7). As a consequence, after learning, the affordance predictions of such objects were inaccurate (far from 0) (object numbers 4, 5 and 6) and showed high variance for most objects (Supplementary Figure 8). This result can be explained by the fact that the predictions for the novel objects are bootstrapped from previously learned affordances of similar objects, in particular based on the color that causes synergies when it involves objects with same present/absent affordance, and interference in the opposite case. A mechanism of replay of past experience would possibly overcome this problem as it would intermix experience related to the different objects, allowing the neural-network predictors to disentangle the present/absent affordances of similar objects.

During the extrinsic phase (Table 6), all the three systems successfully accomplished the goal in the first three scenarios but not in the last two.

**Table 6:** Third late-object test: success of the three systems IGN, FIX, and IMP in the five extrinsic scenarios.

	**Extrinsic-phase scenario**				
**System**	**1**	**2**	**3**	**4**	**5**
IGN	0.8	0.8	0.7	0.0	0.0
FIX	0.9	0.9	0.8	0.0	0.0
IMP	1.0	0.9	0.8	0.0	0.0

*Data as in Table 2*.

### 3.2. Stochastic Environment

#### 3.2.1. Learning of Stochastic Affordances

The stochastic environment used stochastic affordances for some objects and actions whereas the other affordances were as in the deterministic environment (Table 7).

**Table 7:** Stochastic environment: affordance probabilities for all objects and actions.

	**Object**	**Move action prob**.	**Turn green prob**.	**Turn red prob**.	**Turn blue prob**.
1	Red square	0.6	0.7	0.0	0.8
2	Green square	1.0	0.0	1.0	0.8
3	Blue square	1.0	0.7	1.0	0.0
4	Red circle	0.6	0.7	0.0	0.8
5	Green circle	1.0	0.0	1.0	0.8
6	Blue circle	1.0	0.7	1.0	0.0
7	Red rectangle	0.6	0.7	0.0	0.8
8	Green rectangle	1.0	0.0	1.0	0.8
9	Blue rectangle	1.0	0.7	1.0	0.0

The intrinsic phase was run 10 times each for 10,000 steps. The plots show the average performance over 10 trials. The leaky average of the maximum utility estimation was updated with a leak rate ν = 0.1 in both the IGN and IMP systems. The learning rate of the affordance predictors was set to α = 0.001 and the learning rate of the learning-progress predictors was set to α = 0.0005.

**Figure 11:**
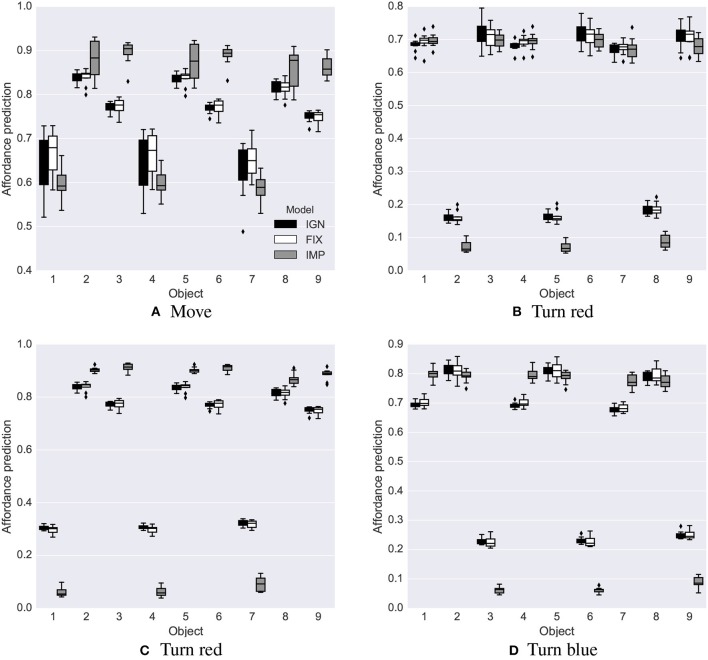
Stochastic environment: affordance predictions after learning. Plotted as in Figure 5. **(A)** Move. **(B)** Turn red. **(C)** Turn red. **(D)** Turn blue.

After learning, all three systems showed a good capacity to predict the affordances, but the IMP system was more accurate than the IGN and FIX systems as it could better employ the available learning time to accumulate more knowledge (Figure 11). In particular: (a) it learned better to estimate affordances, with a probability equal to 1.0 (Figures 11A,C) or 0.0 (Figures 11B–D); (b) it correctly learned the 0.8 probability for the change-color-to-blue action (Figure 11D).

Both the FIX and IGN systems fail to learn accurate affordance probabilities as they get high motivation signals for exploring stochastic objects even when there is no more knowledge to be gained on them. Only the IMP system is able to focus on different objects depending on the actual learning progress they can furnish.

As the IMP system learned affordance probabilities better than IGN and FIX, we ran the extrinsic-phase tests illustrated in the next section using only such a system.

#### 3.2.2. Goal-Based Planning vs. Utility-Based Planning

The goal-based planner and the utility-based planner were compared by running an extrinsic-phase test in a stochastic environment with the four objects indicated in Table 8. The four objects were assigned different values and the actions required for accomplishing each sub-goal had different probabilities of success (those corresponding to the affordances learned in the intrinsic phase). This resulted in a different expected utility of the objects.

**Table 8:** Utility planning test: Objects in the utility planning test and their corresponding values, probability of goal-accomplishing action success and expected utility.

**Object**	**Value**	**P(action success)**	**Expected utility**
Blue circle	1	0.7	0.7
Green square	1	0.8	0.8
Red rectangle	2	0.7	1.4
Red square	4	0.6	2.4

The test was run 20 times for each of the 10 simulations of the intrinsic phase using different action budgets available to the system (1 to 5 actions). A small action-budget constraint introduces the need of deciding which actions to perform based on their expected utility.

**Figure 12:**
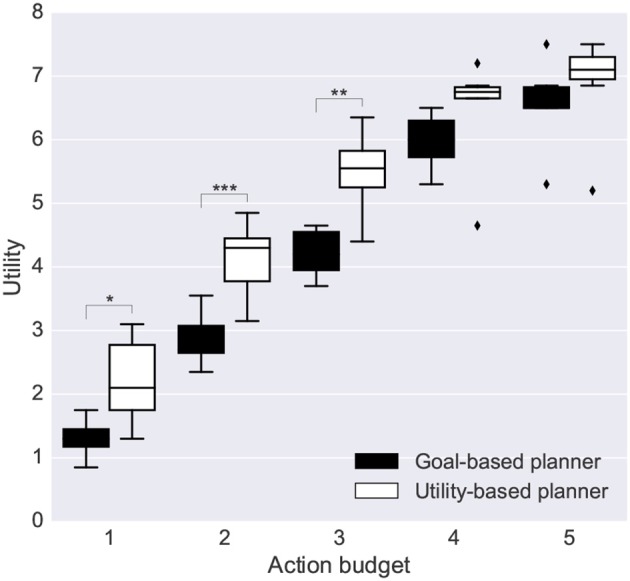
Performance of the goal-based planner and the utility-based planner. Each bar is the average utility over 10 repetitions of the intrinsic-phase training and 20 runs of the extrinsic-phase test. Statistical significance was computed with a double-tailed *t*-test (Mid-line of boxes shows median values, boxes show quartiles, and bars show the min-max range, of the intrinsic-phase repetitions): ^*^p < 0.05; ^**^p < 0.01; and ^***^p < 0.001.

The results show that the utility-based planner performed significantly better than the goal-based planner when it could rely on a small number of actions, and showed a statistical trend to do so for a higher number of actions (Figure 12). This shows an advantage of affordances for planning when the system knows the utility of different alternative sub-goals.

The smaller difference between the models in utility for a higher number of actions is expected due to the fact that if all goals can be accomplished, independently of their utility, the order of their accomplishment does not matter. However, reality, offering a very large number of alternative (sub-)goals with respect to the actions that can be performed, is similar to the case of the experiment where the system has only 1 or 2 actions available, so utility-based planning is very important in such conditions.

#### 3.2.3. Learning of Forward Models in the Stochastic Environment

**Figure 13:**
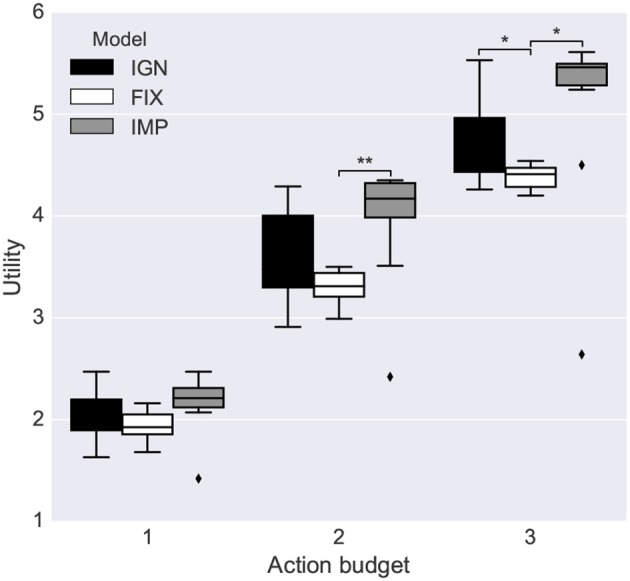
Stochastic environment: quality of forward models acquired. Statistical significance is based on a double-tailed *t*-test (Mid-line of boxes shows median values, boxes show quartiles, and bars show the min-max range, of the ten intrinsic-phase repetitions). ^*^p < 0.05; ^**^p < 0.01.

Having illustrated the utility-planning experiment, it is now possible to show that IMP outperformed IGN and FIX not only in terms of the quality of learned affordances but also in terms of the quality of the learned forward models. To this purpose, we compared the performance of the utility-planner using affordances and forward models trained with either one of the IGN/FIX/IMP mechanisms for 4,000 executed actions, a time not sufficient to fully learn the forward models. Figure 13 shows the performance (overall gained utility) of the three utility planners using a maximum of 1, 2, or 3 actions and averaged over 100 repetitions of the experiment. The results show that IMP has a higher performance than IGN and FIX in all conditions; in particular, it is statistically better than FIX with 2 and 3 actions (*p* < 0.05), and better than IGN with 3 actions (*p* < 0.05).

The better performance of IMP could be due to either worse affordances or worse forward models of IGN and FIX. To ascertain this, we repeated the experiment using forward models trained for a time allowing convergence (10,000 executed actions) for all the three systems. In this case the three systems showed a similar performance (data not reported). This indicates that the better performance of IMP in the previous experiment was due to better forward models. A possible explanation of this is that there is a correlation between the difficulty of learning the predictors estimating the affordance-probabilities and the predictors implementing the what-effect forward models as they share the same input (object image). So the effective decisions of IMP on which experiences to focus on to learn affordances also benefit the learning of the forward models. On the other hand, even if affordances of IGN and FIX have a lower quality than IMP (section 3.2.1), this does not negatively affect their performance as such lower quality does not impair the utility-based ranking of the object-related sub-goals.

#### 3.2.4. Affordances Allow the Reduction of the Forward Planning Search

**Figure 14:**
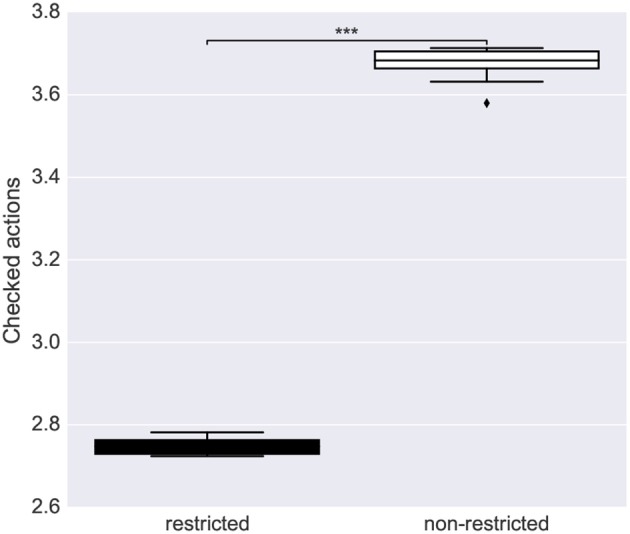
Average number of actions to check for accomplishing each sub-goal in the case of affordance-based restricted and nonrestricted planning search. Statistical significance is based on a double-tailed t-test (Mid-line of boxes shows median values, boxes show quartiles, and bars show the min-max range, of the intrinsic-phase successful repetitions). ^***^p < 0.001.

We have seen that a potential benefit of affordances for planning is the possibility of reducing the number of actions that should be checked during the generation of the forward trajectories. To validate this idea, we ran again the previous extrinsic-phase test (section 3.2.2) but without constraining the number of actions that the system could perform. In particular, we compared two systems, a first one checking all available actions and a second one restricting the forward-model-based search to only those actions having an affordance ≥ 0.5 (here this value excludes from the search all non-afforded actions). The results show that the use of affordances allows a significant reduction of the mean number of actions checked (Figure 14).

Consider that in realistic situations, the number of actions that can be performed in a certain condition is very high. Moreover, often several actions can be performed in sequences to accomplish a certain (sub-)goal, a situation not investigated here. In this case, the possible reduction based on affordances of the branching factor due to actions is even more important.

## 4. Other Relevant Models in the Literature

The architecture presented here integrates functionalities that have been investigated in isolation in other computational systems. In this section we review the systems that are more closely related to the system presented here, and compare their main features.

Many works have focused on intrinsic motivations as a means to solving extrinsic challenging tasks where a long sequence of skills is required to solve a task or maximize a specific reward function (“sparse reward,” e.g., Santucci et al., 2014). Instead, fewer works on open-ended learning have focused on intrinsic motivations for the autonomous acquisition of skills that are assembled in sequences in a later “extrinsic phase.” For example, Schembri et al. (2007) and Kulkarni et al. (2016) present two reinforcement learning systems that undergo two separated learning phases. In the second extrinsic phase, both systems use reinforcement learning to solve complex tasks by assembling sequences of skills acquired in the first phase. In the intrinsic phase, the first system learns the skills on the basis of reinforcement learning guided by intrinsic motivations and reward functions found by a genetic algorithm that uses the performance in the extrinsic phase as fitness. Instead, during the intrinsic phase the second system learns the skills on the basis of a mechanism generating skills when the agent's action causes “two objects to interact.” Although these systems employ the idea of the two phases to develop and test open-ended learning systems, they do not investigate how they could learn affordances in the intrinsic phase and their possible use for planning.

Seepanomwan et al. (2017) investigates how a robot can exploit knowledge acquired with open-ended learning in an intrinsic phase to accomplish user-defined goals in a later extrinsic phase. In a first intrinsic phase the robot autonomously generates multiple “goals/outcomes” related to moving a ball in different positions on a table. In a following extrinsic phase the system reuses the acquired goals and skills to directly accomplish new goals (assigned to it by a user) or to more quickly learn the skills to do so. To this purpose, the system performs a one-step backward planning search by searching for the best skill to perform on the basis of the similarity of the user's goal with the goals of all skills. Contrary to here, the system can solve only simple but not compound tasks, can interact with only one object at a time since it does not have an attention system, and the initial condition is identical in each trial (the environment is “reset” after each skill performance).

Planning represents a central theme in artificial intelligence (Ghallab et al., 2004; Russell and Norvig, 2016). Here we only considered few aspects of planning relevant to face the issues related to open-ended learning and exploitation of affordances. Investigating the possible roles of affordances with planning, we have seen that when forward planning is used affordances allow the agent to short-list the available actions. Interestingly (cf. Russell and Norvig, 2016), until the late 90's the research on planning mainly focused on backward planning because this revealed more efficient than forward planning generating a wide search branching as many actions are applicable to each state. Later, forward planning became popular again, thanks to general-purpose heuristics that allowed the reduction of the search breadth based on domain-independent general heuristics, for example focusing only on the “positive effects” (usually denoting the action success) while ignoring the “delete effects” (usually involved in the violation of other sub-goals). This suggests that the common use of forward planning by organisms (Wikenheiser and Redish, 2015) might rely on affordances for pruning relevant actions: affordances hence are so important for organisms (Thill et al., 2013) because they not only support an efficient action but also planning.

The work presented here has multiple links with the autonomous/developmental robotics literature on affordances. In an early work, Stoytchev (2005) was among the first to propose the idea of a robot using affordances related to different objects (in this case tools) to evaluate their effects. Affordances, stored in a table, regarded the effects that actions could produce on different tools. Among other things, the work established the importance of focusing on objects rather than on the whole “state” of the environment for processing affordances.

Regarding the link between affordance acquisition and open-ended learning, Ugur et al. (2007) was among the first to use intrinsic motivations to support the acquisition of affordance knowledge. In particular, it investigated a mobile robot that had to learn to evaluate the “traversability” of a set of obstacle-objects in front of it. The robot scanned different possible directions of movement and decided to attempt to move along one of them if its ignorance with respect to the possible success in doing so was above a certain threshold. This aimed to invest the time and energy of the agent on learning the more uncertain affordances. Here we compared this mechanism with a more sophisticated mechanism where the ignorance for the current object and more uncertain affordance is compared with the estimated average ignorance for the other objects and affordances on which exploration time and energy might be alternatively invested.

The link between affordances and the possible relations between the elements of the object/action/effect triad was investigated by Montesano et al. (2008). The work equated affordances with the relations between such three elements and represented them with a Bayesian network within a probabilistic framework. Here we assumed a restricted definition of affordances, more closely linked to the initial definition, and this allowed us to investigate their relations with the elements involved in planning. Building on the probabilistic framework of the work by Montesano et al. (2008), Gonçalves et al. (2014) propose to model affordances as the interaction between tools and objects based on their physical (geometrical) properties.

The link between affordances and planning was investigated in Ugur et al. (2009, 2011). The authors equated affordances to the forward models, in particular to the triad <object-features, action, effect>, where actions are pre-coded behaviors for moving or lifting objects and effects are clustered with a support vector machine. In a first phase the system learns the affordances and in a second phase the system is assigned a goal and plans the course of actions to pursue it based on a breadth-first forward search over actions and states until it finds a state similar to the goal. In Mar et al. (2015) a robot explored a pulling action performed with a rake-like tool to retrieve a target object. This allowed the robot to learn a mapping (through a support vector machine) between the pose of the tool in space and the affordances intended as the class (produced with a k-means clustering) of the possible “action parameters-retrieval effect” concatenated feature vector. For a given tool pose, this allowed the robot to select related affordance (action-effect class) and then to select the action parameters corresponding to the highest effect. This system shares some resemblance with the utility planner used here with the difference that in Mar et al. (2015) affordances support the selection of actions based on the amount of the expected desired (continuous) effect, whereas in our system they support the selection of actions based on their probability of producing the desired effect (which can be present/absent).

The affordance concept used here is analogous to the one of “preconditions” used in STRIPS-based planning *operators*. Preconditions establish if the operator is applicable or not to the current environment state (Fikes and Nilsson, 1972; Russell and Norvig, 2016). A similar concept is the “initiation set” of *options* in the reinforcement learning literature. The initiation set encompasses the states in which the action policy of the option can be performed (Sutton et al., 1999). Both concepts are deterministic. Instead, in utility-based reasoning actions produce desired effects only with a certain probability (Russell and Norvig, 2016). We think this action-success probability is the concept of artificial intelligence that is more similar to the concept of affordance used here.

Some of the relations between attention, affordances and intrinsic motivations were investigated in Nguyen et al. (2013) and Ivaldi et al. (2014). They proposed a robotic system endowed with a bottom-up attention system to detect the various objects available in the scene. Intrinsic motivations related to knowledge acquisition (3D object recognition) were used by the robot to decide which strategy to use (autonomous vs. social) to decide on which object to focus the learning resources. The work did not investigate, as here, the specific effects of a restricted focus of attention on the intrinsic-motivation mechanisms supporting affordance learning.

A last field of research related to this work involves active vision (Ballard, 1991). This literature is relevant as many action affordances tend to involve objects and a controllable visual sensor with a limited perceptual scope is a means to isolate information on specific objects (not considering here the important problem related to the fact that objects have different sizes and this requires an adjustable visual scope). Previous works (Ballard, 1991; Ognibene and Baldassare, 2015) have shown how such systems decrease the computational burden required by processing too wide visual images, and research on state-of-the-art deep neural networks applied to vision problems is confirming the utility of an attentional focus (Xu et al., 2015). Here we use active vision in a different way to extract information on single objects, factored into information about the object state and its location in space. As mentioned in the introduction, this is a fundamental operation representing a first important step from a *factored* (featured-based) representation of the world state to a *structured* representation, allowing to reason on the state and relations between objects, typically used in classic planning (Russell and Norvig, 2016). Although this does not still allow the performance of the complex logic-based reasoning of classical planning, it allows the parsing of the whole goal into specific solvable object-centered sub-goals in the extrinsic phase.

We started to explore the factorization of a scene by an active vision system endowed with controllable restricted visual sensors in a camera-arm robot interacting with simple-shaped 2D objects as those used here (Ognibene et al., 2008, 2010). The sensor of this system was controlled not only with a bottom-up attention component, as here, but also by a top-down component able to learn to find a desired target-object by reinforcement learning: the latter component might be integrated into the current model in the future. The system was developed to accomplish only one extrinsic task, rather than multiple ones as here, and did not deal with open-ended learning. A system developed starting from the previous ones (Sperati and Baldassarre, 2014), again endowed with a bottom-up and a top-down attention component, is instead able to self-generate and learn tasks based on intrinsic motivations. This system has been further developed to self-generate and encode multiple visual-target *goals*, and to learn the camera-controller to find them (Sperati and Baldassarre, 2018). These systems have functionalities complementary with those of the system presented here, so they might be suitably integrated in the future to have a system able to: (a) self-generate goals and use them to drive the learning of the attention and motor skills to accomplish them through intrinsic motivations (previous systems) and (b) develop affordances and re-use the previously acquired skills to solve complex extrinsic tasks (system proposed here).

## 5. Conclusions and Future Work

This work has focused on a possible specific instance of the concept of affordance intended as the probability of achieving a certain desired outcome associated to an action, by performing such action on a certain object. We investigated here three issues related to this concept: (a) within an open-ended autonomous learning context, how can intrinsic motivations guide affordance learning in a system that moves the attention of a visual sensor over different objects; (b) how can such an attention process support the decomposition of complex goals (tasks), involving multiple objects, into separated sub-goals related to single objects; (c) what could be the added value of affordances in planning systems already having sophisticated forward models of the world. For each issue we presented possible advancement with respect to the state of the art (section 4), and showed their advantages in specific experiments (section 3). Several aspects of the system could however be improved in future work.

Regarding the first issue, we proposed a mechanism to use intrinsic motivations (system IGN) to improve previously proposed ways (Ugur et al., 2007) with which a system endowed with a mechanism focusing on only one object/condition per time can decide whether or not to invest energy to explore it. The proposed mechanism is quite general and could in principle be used for any decision making process supported by a collection of information from the environment based on selective attention. The proposed solution is based on an adjustable variable storing the *opportunity cost* of the current choice, i.e., the value that the system looses by selecting the current option rather than alternative ones (Buchanan, 2008). The presented experiments support the effectiveness of this mechanism.

With respect to intrinsic motivations, in the case of deterministic scenarios where the system knows in advance that the affordance probability is either 0 or 1, the value of actions on the current object and the cost of alternatives was here estimated in terms of intrinsic motivations measuring the system ignorance (system IGN). This is not possible in stochastic scenarios where the affordance probability can be any value ranging in (0, 1), so we proposed an intrinsic motivation tied to the improvement, rather than the level, of the probability estimation (system IMP). This solution, building on previous works on intrinsic motivations (e.g., Schmidhuber, 1991a; Santucci et al., 2013), led to a faster learning of affordance probabilities in our tests. However, an open problem of this solution, known in the literature (Santucci et al., 2013), is the fact that *error improvement* signals, as those used to compute intrinsic motivation in IMP, are small with respect to noise as they are equivalent to a derivative in time (vs. *error* signals used by IGN-like systems). This makes them more unstable: future work should face this problem.

Another important aspect related to autonomous learning driven by intrinsic motivations is that here, for the sake of focussing the research, the current system learns affordances on the basis of pre-wired actions and goals (expected outcomes of affordances). In a fully autonomous open-ended learning agent such actions and goals should instead be autonomously learned. Much literature has focused on the autonomous learning of actions and, more recently, of goals (e.g., Kulkarni et al., 2016; Santucci et al., 2016; Forestier et al., 2017; Cartoni and Baldassarre, 2018; Nair et al., 2018). Future work should thus aim to integrate the autonomous learning of affordances with the autonomous learning of actions and goals.

Regarding the second issue, related to the advantage for planning of having an attention system focusing on objects, we showed how the parsing of the scene into objects allows the solution of non-trivial planning problems on the basis of relatively simple one-step planning mechanisms. This agrees with previous proposals, such as the “object action compound” framework (Krüger et al., 2011; see also Montesano et al., 2008) stating the importance of representing information based on objects. Future work should investigate the advantage of object-centered attention and *multi-step* planning.

Although the introduction of focused visual sensors (attention) facilitates the parsing of the scene into objects, it also makes decision making more difficult. Indeed, the system has to look at different objects, and store information on them, to decide on which object to act or not. We have seen that the information to store can for example involve either the expected information gain, as requested by intrinsic motivations, or the utility of sub-goals, as requested by the solution of a utility-based problem. Here we have proposed a first solution to this problem that requires low computational resources (scanning objects in sequence, computing their expected utility, updating a variable that stores the maximum expected utility encountered this far, and deciding to act on the current object depending on how its utility compares with the maximum expected utility). This mechanism proved effective in tests. However, other more efficient (but also computationally more expensive) mechanisms could be used, in particular based on a memory of the specific utility of the different scanned objects. This information could be indexed by the different positions that have been visually inspected in the scene so that the information itself is readily usable to guide top-down attention processes and actions on specific target objects (Ognibene and Baldassare, 2015).

Regarding the third and last issue, related to the possible added value of affordances in planning systems, we showed that affordances as defined here can be useful in goal-based planning systems as they allow a search focused on actions that can be used in the current context. In section 4 we mentioned that this function is similar to that played by preconditions in STRIPS-based planning and by the “initiation set” in reinforcement-learning options. We have also seen that a second function that affordances can play, in particular for utility-based planning problems, is for weighting the importance of alternative goals based on the probability to accomplish them. The definition used to this purpose, $Pr\left(s_{b,o}^{\prime }\in G|a,s_{b,o}\right)$ can be related to one of *internal models* of the *transition function Pr*(*s*′|*a, s*) used in model-based reinforcement-learning systems (Sutton and Barto, 2018). These models take into consideration stochastic environments rather than deterministic ones, as usually done in symbolic planning; but on the other hand they use *atomic representations*, *s*, of whole states, rather than information on single objects and their relations as done in symbolic planning using *structured representations* (Russell and Norvig, 2016). In this respect, the concept of affordances used here, pivoting on *s*_*b, o*_, starts to integrate the two approaches as it focuses on single objects (the body and the target object), and at the same time it considers probabilities of their states (specifically encoded with *factored representations*, such as pixels images, as commonly done in robotic reinforcement learning models, Wiering and Van Otterlo, 2012). Future work could further develop this integration (e.g., see Konidaris et al., 2018).

Overall, we think that showing how attention can support a representation of information centered on objects rather than on whole states, and the implications of this for autonomous affordance learning and planning, is a very important issue to which this work contributed.

We conclude by discussing how the system might scale up to more complex scenarios. The overall architecture is expected to scale up well to more complex environments but the implementation of its components should be enhanced to such purpose. For the sake of simplicity here we developed the model components in a way that was sufficient to tackle a simple environment featuring a black background and non-overlapping objects. A realistic environment with a rich texture and several possibly-overlapping objects would produce cluttered images. To face this condition the system should be endowed with object segmentation capabilities (Zhang et al., 2008) or robust object recognition algorithms such as deep neural networks (LeCun et al., 2015). Interestingly, some of these latter algorithms have started to use attention mechanisms to improve object recognition (Maiettini et al., 2018): future work might investigate the links between these mechanisms and the attention processes presented here. More powerful deep learning models might also be used to implement the predictors used in the architecture. A last critical component is the simple bottom-up attention mechanism used to identify objects, and, as expected, this was limited (it scaled worse than linearly with the number of objects). The component could be enhanced with the addition of more sophisticated top-down attention mechanisms able to drive attention on the basis of the current knowledge on the identity and position of objects in the scene (Rasolzadeh et al., 2010; Sperati and Baldassarre, 2014, 2018; Ognibene and Baldassare, 2015).

A final general feature of the system that should be addressed in future work is the fact that the information flows between the several components of the architecture are managed by a hard-coded central algorithm using time flags and in some cases symbolic representations. This feature is shared by most architectures of this type. An alternative approach would be to follow the design of real brains where the information flows between components is continuous and has a distributed nature. An example of this is given in Baldassarre et al. (2013) proposing an architecture for goal-based open-ended learning where components are implemented on the basis of leaky-neuron neural networks. This strategy has the advantage of a higher biological realism (relevant when brain modeling is the research objective) and for having a higher tolerance to noise affecting the timing of events. On the other side it has the disadvantages of making it more difficult to tune the whole system and also to use some learning algorithms that require a precise timing of events.

## Author Contributions

GB: overall idea of the system, specification of the model and tests, analysis of results, and writing-up. WL: specification of the model and tests, implementation of the system, tests, data analysis, analysis of results, and writing-up. GG: specification of the model and initial tests, and analysis of results. VS: specification of the model and tests, analysis of results, and writing-up.

### Conflict of Interest Statement

The authors declare that the research was conducted in the absence of any commercial or financial relationships that could be construed as a potential conflict of interest.

## Footnotes

^1^This and more complex versions of the scenario involving autonomous robots have been developed within the EU funded project “GOAL-Robots – Goal-based Open-ended learning Autonomous Robots,” www.goal-robots.eu.

## References

[B1] Asada M. MacDorman K. F. Ishiguro H. Kuniyoshi Y. (2001). Cognitive developmental robotics as a new paradigm for the design of humanoid robots. Robot. Auton. Syst. 37, 185–193. 10.1016/S0921-8890(01)00157-9

[B2] Baldassarre G. (2002). Planning with neural networks and reinforcement learning. (Ph.d. thesis). Computer Science Department, University of Essex, Colchester, United Kingdom.

[B3] Baldassarre G. (2011). What are intrinsic motivations? A biological perspective, in Proceedings of the International Conference on Development and Learning and Epigenetic Robotics (ICDL-EpiRob-2011), 24-27 August (Frankfurt am Main), E1–E8.

[B4] Baldassarre G. Mannella F. Fiore V. G. Redgrave P. Gurney K. Mirolli M. (2013). Intrinsically motivated action-outcome learning and goal-based action recall: a system-level bio-constrained computational model. Neural Netw. 41, 168–187. 10.1016/j.neunet.2012.09.015 23098753

[B5] Baldassarre G. Mirolli M. (2013b). Intrinsically motivated learning systems: an overview, in Intrinsically Motivated Learning in Natural and Artificial Systems, eds Baldassarre G. Mirolli M. (Springer-Verlag, Berlin), 1–14.

[B6] Baldassarre G. Mirolli M. (eds.). (2013a). Intrinsically Motivated Learning in Natural and Artificial Systems. Berlin; Heidelberg: Springer.

[B7] Ballard D. H. (1991). Animate vision. Artif. Intell. 48, 57–86. 10.1016/0004-3702(91)90080-4

[B8] Baranes A. Oudeyer P. (2013). Active learning of inverse models with intrinsically motivated goal exploration in robots. Robot. Auton. Syst. 61, 49–73. 10.1016/j.robot.2012.05.008

[B9] Barto A. Mirolli M. Baldassarre G. (2013). Novelty or surprise? Front. Psychol. 4:907 10.3389/fpsyg.2013.00907 24376428 PMC3858647

[B10] Barto A. G. Singh S. Chentanez N. (2004). Intrinsically motivated learning of hierarchical collections of skills, in International Conference on Developmental Learning (ICDL2004) (La Jolla, CA), 112–119.

[B11] Bratman M. E. (1987). Intentions, Plans, and Practical Reason. Cambridge, MA: Harvard University Press.

[B12] Buchanan J. M. (2008). Opportunity Cost, 2nd Edn. London, UK: Macmillan Publishers.

[B13] Camoriano R. Pasquale G. Ciliberto C. Natale L. Rosasco L. Metta G. (2017). Teaching robots to learn new objects in constant time. arXiv:1605.05045v2.

[B14] Cartoni E. Baldassarre G. (2018). Autonomous discovery of the goal space to learn a parameterized skill. arXiv 1805.07547v1.

[B15] Castellini C. Tommasi T. Noceti N. Odone F. Caputo B. (2011). Using object affordances to improve object recognition. IEEE Trans. Auton. Ment. Dev. 3, 207–215. 10.1109/TAMD.2011.2106782

[B16] Comoli E. Coizet V. Boyes J. Bolam J. P. Canteras N. S. Quirk R. H.. (2003). A direct projection from superior colliculus to substantia nigra for detecting salient visual events. Nat. Neurosci. 6, 974–980. 10.1038/nn1113 12925855

[B17] Dauce E. (2018). Fovea-based scene decoding through computationally-effective model-based prediction. Front. Neurorobot. 12:76 10.3389/fnbot.2018.00076 30618705 PMC6302111

[B18] Fikes R. E. Nilsson N. J. (1972). Strips: A new approach to the application of theorem proving to problem solving. Artif. Intell. 2, 189–208. 10.1016/0004-3702(71)90010-5

[B19] Fitzpatrick P. Metta G. Natale L. Rao S. Sandini G. (2003). Learning about objects through action-initial steps towards artificial cognition, in Proceedings of the IEEE International Conference on Robotics and Automation (ICRA03), Vol. 3 (Taipei: IEEE), 3140–3145.

[B20] Forestier S. Mollard Y. Oudeyer P.-Y. (2017). Intrinsically motivated goal exploration processes with automatic curriculum learning. arXiv 1708.02190v1.

[B21] Gandhi N. J. Katnani H. A. (2011). Motor functions of the superior colliculus. Annu. Rev. Neurosci. 34, 205–231. 10.1146/annurev-neuro-061010-113728 21456962 PMC3641825

[B22] Ghallab M. Nau D. Traverso P. (2004). Automated Planning: Theory and Practice. Amsterdam: Elsevier.

[B23] Gibson J. J. (1979). The Ecological Approach to Visual Perception. Boston, MA: Houghton Mifflin.

[B24] Gonçalves A. Saponaro G. Jamone L. Bernardino A. (2014). Learning visual affordances of objects and tools through autonomous robot exploration, in Autonomous Robot Systems and Competitions (ICARSC), 2014 IEEE International Conference on (Espinho: IEEE), 128–133.

[B25] Ivaldi S. Lyubova N. Droniou A. Padois V. Filliat D. Oudeyer P.-Y. (2014). Object learning through active exploration. IEEE Trans. Auton. Ment. Dev. 6, 56–72. 10.1109/TAMD.2013.2280614

[B26] Konidaris G. Kaelbling L. P. Lozano-Perez T. (2018). From skills to symbols: learning symbolic representations for abstract high-level planning. J. Artif. Intell. Res. 61, 215–289. 10.1613/jair.5575

[B27] Korf R. E. (1985). Macro-operators: a weak method for learning. Artif. Intell. 26, 35–77. 10.1016/0004-3702(85)90012-8

[B28] Krüger N. Geib C. Piater J. Petrick R. Steedman M. Wörgötter F. (2011). Object–action complexes: grounded abstractions of sensory–motor processes. Robot. Auton. Syst. 59, 740–757. 10.1016/j.robot.2011.05.009

[B29] Kulkarni T. D. Narasimhan K. R. Saeedi A. Tenenbaum J. B. (2016). Hierarchical deep reinforcement learning: integrating temporal abstraction and intrinsic motivation. arXiv 1604.06057.

[B30] LeCun Y. Bengio Y. Hinton G. (2015). Deep learning. Nature 521, 436–444. 10.1038/nature14539 26017442

[B31] Lungarella M. Metta G. Pfeifer R. Sandini G. (2003). Developmental robotics: a survey. Connect. Sci. 15, 151–190. 10.1080/09540090310001655110

[B32] Maiettini E. Pasquale G. Rosasco L. Natale L. (2018). Speeding-up object detection training for robotics with falkon. arXiv:1803.08740. 10.1109/IROS.2018.8593990

[B33] Mar T. Tikhanoff V. Metta G. Natale L. (2015). 2d and 3d functional features for tool affordance learning and generalization on humanoid robot, in Proceedings of the IEEE/RSJ International Conference on Intelligent Robots and Systems Workshop Learning Object Affordances Fundamental Step Allow Prediction Planning Tool Use (Hamburg).

[B34] Mirolli M. Baldassarre G. (2013). Functions and mechanisms of intrinsic motivations: the knowledge versus competence distinction, in Intrinsically Motivated Learning in Natural and Artificial Systems, eds Baldassarre G. Mirolli M. (Berlin: Springer-Verlag), 49–72.

[B35] Montesano L. Lopes M. Bernardino A. Santos-Victor J. (2008). Learning object affordances: from sensory–motor coordination to imitation. IEEE Trans. Robot. 24, 15–26. 10.1109/TRO.2007.914848

[B36] Nair A. Pong V. Dalal M. Bahl S. Lin S. Levine S. (2018). Visual reinforcement learning with imagined goals, in The Second Lifelong Learning: A Reinforcement Learning Approach Workshop (LLRLA2018 at FAIM2018), number 1807.04742. Stockholm.

[B37] Nguyen S. M. Ivaldi S. Lyubova N. Droniou A. Gerardeaux-Viret D. Filliat D. (2013). Learning to recognize objects through curiosity driven manipulation with the icub humanoid robot, in IEEE International Conference on Development and Learning-Epirob (Osaka).

[B38] Ognibene D. Baldassare G. (2015). Ecological active vision: four bio-inspired principles to integrate bottom-up and adaptive top-down attention tested with a simple camera-arm robot. IEEE Trans. Auton. Ment. Dev. 7, 3–25. 10.1109/TAMD.2014.2341351

[B39] Ognibene D. Balkenius C. Baldassarre G. (2008). Integrating epistemic action (active vision) and pragmatic action (reaching): a neural architecture for camera-arm robots, in From Animals to Animats 10: Proceedings of the Tenth International Conference on the Simulation of Adaptive Behavior (SAB2008), volume 5040 of *Lecture Notes in Artificial Intelligence, Osaka, Japan, 7-12 July 2008*, eds Asada M. Hallam J. C. Meyer J.-A. Tani J. (Berlin: Springer Verlag), 220–229.

[B40] Ognibene D. Pezzulo G. Baldassarre G. (2010). How can bottom-up information shape learning of top-down attention-control skills?, in Proceedings of 9th IEEE International Conference on Development and Learning (ICDL2010) (Ann Arbor, MA), 231–237.

[B41] Oudeyer P.-Y. Kaplan F. Hafner V. V. (2007). Intrinsic motivation systems for autonomous mental development. IEEE Trans. Evol. Comput. 11, 265–286. 10.1109/TEVC.2006.890271

[B42] Oudeyer P. Y. Kaplan F. (2007). What is intrinsic motivation? A typology of computational approaches. Front. Neurorobot. 1:6. 10.3389/neuro.12.006.2007 18958277 PMC2533589

[B43] Rasolzadeh B. Björkman M. Huebner K. Kragic D. (2010). An active vision system for detecting, fixating and manipulating objects in the real world. Int. J. Robot. Res. 29, 133–154. 10.1177/0278364909346069

[B44] Russell S. J. Norvig P. (2016). Artificial Intelligence: A Modern Approach, 3rd Edn. Harlow: Pearson.

[B45] Ryan R. M. Deci E. L. (2000). Intrinsic and extrinsic motivations: classic definitions and new directions. Contemp. Educ. Psychol. 25, 54–67. 10.1006/ceps.1999.1020 10620381

[B46] Santucci V. G. Baldassarre G. Mirolli M. (2012). Intrinsic motivation mechanisms for competence acquisition, in Proceeding of the IEEE International Conference on Development and Learning and Epigenetic Robotics (ICDL-EpiRob 2012), 7-9 November 2012 (San Diego, CA: IEEE), 1–6.

[B47] Santucci V. G. Baldassarre G. Mirolli M. (2013). Which is the best intrinsic motivation signal for learning multiple skills? Front. Neurorobot. 7:22. 10.3389/fnbot.2013.00022 24273511 PMC3824099

[B48] Santucci V. G. Baldassarre G. Mirolli M. (2014). Cumulative learning through intrinsic reinforcements, in Evolution, Complexity and Artificial Life, eds Cagnoni S. Mirolli M. Villani M. (Berlin: Springer), 107–122.

[B49] Santucci V. G. Baldassarre G. Mirolli M. (2016). Grail: a goal-discovering robotic architecture for intrinsically-motivated learning. IEEE Trans. Cogn. Dev. Syst. 8, 214–231. 10.1109/TCDS.2016.2538961

[B50] Schembri M. Mirolli M. Baldassarre G. (2007). Evolving internal reinforcers for an intrinsically motivated reinforcement-learning robot, in Proceedings of the 6th IEEE International Conference on Development and Learning (ICDL2007) (Piscataway, NJ), 282–287.

[B51] Schmidhuber J. (1991a). A possibility for implementing curiosity and boredom in model-building neural controllers, in Proceedings of the International Conference on Simulation of Adaptive Behavior: From Animals to Animats. eds Meyer J. Wilson S. (Boston, MA: MIT Press).

[B52] Schmidhuber J. (1991b). Curious model-building control systems, in Proceedings of the International Joint Conference on Artificial Neural Networks, Vol. 2 (Sydney, NSW), 1458–1463.

[B53] Seepanomwan K. Santucci V. G. Baldassarre G. (2017). Intrinsically motivated discovered outcomes boost user's goals achievement in a humanoid robot, in The Seventh Joint IEEE International Conference on Development and Learning and on Epigenetic Robotics (ICDL-EpiRob2017), eds Santos-Victor J. Sandini G. (Lisbon: Istituto Superior Technico), 178–183.

[B54] Sperati V. Baldassarre G. (2014). Learning where to look with movement-based intrinsic motivations: a bio-inspired model, in International Conferences on Development and Learning and Epigenetic Robotics (ICDL-Epirob) (Genoa: IEEE), 461–468.

[B55] Sperati V. Baldassarre G. (2018). A bio-inspired model learning visual goals and attention skills through contingencies and intrinsic motivations. IEEE Trans. Cogn. Dev. Syst. 10 10.1109/TCDS.2017.2772908

[B56] Stoytchev A. (2005). Behavior-grounded representation of tool affordances, in Proceedings of the 2005 IEEE International Conference on Robotics and Automation (ICRA 2005) (Barcelona), 3060–3065.

[B57] Sutton R. (1990). Integrated architectures for learning, planning, and reacting based on approximating dynamic programming, in Proceedings of the Seventh International Conference on Machine Learning, Vol. 216 (Austin, TX), 216–224.

[B58] Sutton R. S. Barto A. G. (2018). Reinforcement Learning: An Introduction, 2nd Edn. Cambridge, MA: The MIT Press.

[B59] Sutton R. S. Precup D. Singh S. (1999). Between mdps and semi-mdps: a framework for temporal abstraction in reinforcement learning. Artif. Intell. 112, 181–211. 10.1016/S0004-3702(99)00052-1

[B60] Sweeney J. D. Grupen R. (2007). A model of shared grasp affordances from demonstration, in Humanoid Robots, 2007 7th IEEE-RAS International Conference on (Pittsburgh, PA: IEEE), 27–35.

[B61] Thill S. Caligiore D. Borghi A. M. Ziemke T. Baldassarre G. (2013). Theories and computational models of affordance and mirror systems: an integrative review. Neurosci. Biobehav. Rev. 37, 491–521. 10.1016/j.neubiorev.2013.01.012 23333761

[B62] Thrun S. Mitchell T. (1995). Lifelong robot learning. Robot. Auton. Syst. 15, 25–46. 10.1016/0921-8890(95)00004-Y

[B63] Ugur E. Dogar M. R. Cakmak M. Sahin E. (2007). Curiosity-driven learning of traversability affordance on a mobile robot, in Development and Learning, 2007. ICDL 2007. IEEE 6th International Conference on (Piscataway, NJ: IEEE), 13–18.

[B64] Ugur E. Oztop E. Sahin E. (2011). Goal emulation and planning in perceptual space using learned affordances. Robot. Auton. Syst. 59, 580–595. 10.1016/j.robot.2011.04.005

[B65] Ugur E. Piater J. (2014). Emergent structuring of interdependent affordance learning tasks, in Proceedings of the Fourth Joint IEEE International Conference on Development and Learning and on Epigenetic Robotics (ICDL-EpiRob2014), eds Metta G. Lee M. Fasel I. (New York, NY: Instituto Italiano di Tecnologia (IIT), IEEE), 481–486.

[B66] Ugur E. Sahin E. Oztop E. (2009). Affordance learning from range data for multi-step planning, in Proceedings of the Ninth International Conference on Epigenetic Robotics: Modeling Cognitive Development in Robotic Systems, number 146 in Lund University Cognitive Studies, eds Cañamero L. Oudeyer P.-Y. Balkenius C. (Lund).

[B67] Ungerleider L. G. Haxby J. V. (1994). ‘what’ and ‘where’ in the human brain. Curr. Opin. Neurobiol. 4, 157–165. 10.1016/0959-4388(94)90066-3 8038571

[B68] Weng J. McClelland J. Pentland A. Sporns O. Stockman I. Sur M.. (2001). Autonomous mental development by robots and animals. Science 291, 599–600. 10.1126/science.291.5504.599 11229402

[B69] White R. W. (1959). Motivation reconsidered: the concept of competence. Psychol. Rev. 66:297. 10.1037/h0040934 13844397

[B70] Wiering M. Van Otterlo M. (2012). Reinforcement Learning – State of the Art, Vol. 12 Berlin: Springer.

[B71] Wikenheiser A. M. Redish A. D. (2015). Hippocampal sequences and the cognitive map, in Analysis and Modeling of Coordinated Multi-neuronal Activity, ed Tatsuno M. (Berlin: Springer), 105–129.

[B72] Xu K. Ba J. Kiros R. Cho K. Courville A. Salakhudinov R. (2015). Show, attend and tell: neural image caption generation with visual attention, in International Conference on Machine Learning (Lille), 2048–2057.

[B73] Yilmaz A. Javed O. Shah M. (2006). Object tracking: a survey. ACM Comput. Surv. 38:13 10.1145/1177352.1177355

[B74] Zhang H. Fritts J. E. Goldman S. A. (2008). Image segmentation evaluation: a survey of unsupervised methods. Comput. Vis. Image Understand. 110, 260–280. 10.1016/j.cviu.2007.08.003

