# Supplementary material for: An Embodied Agent Learning Affordances With Intrinsic Motivations and Solving Extrinsic Tasks With Attention and One-Step Planning
Source: Front Neurorobot. 2019 Jul 26;13:45. doi: 10.3389/fnbot.2019.00045 (PMC6676802; doi:10.3389/fnbot.2019.00045)
Supplement: Supplementary file 1 [file Image_1.pdf]

# An embodied agent learning affordances with intrinsic motivations and solving extrinsic tasks with attention and one-step planning SUPPLEMENTARY MATERIAL

**Gianluca Baldassarre** [1,*], **William Lord** [2], **Giovanni Granato** [1], **and Vieri Giuliano Santucci** [1]

[1] *Laboratory of Computational Embodied Neuroscience, Institute of Cognitive Sciences and Technologies, National Research Council of Italy, Rome, Italy*
[2] *School of Engineering Sciences, KTH Royal Institute of Technology, Stockholm, Sweden*

Correspondence*:
Gianluca Baldassarre
gianluca.baldassarre@istc.cnr.it

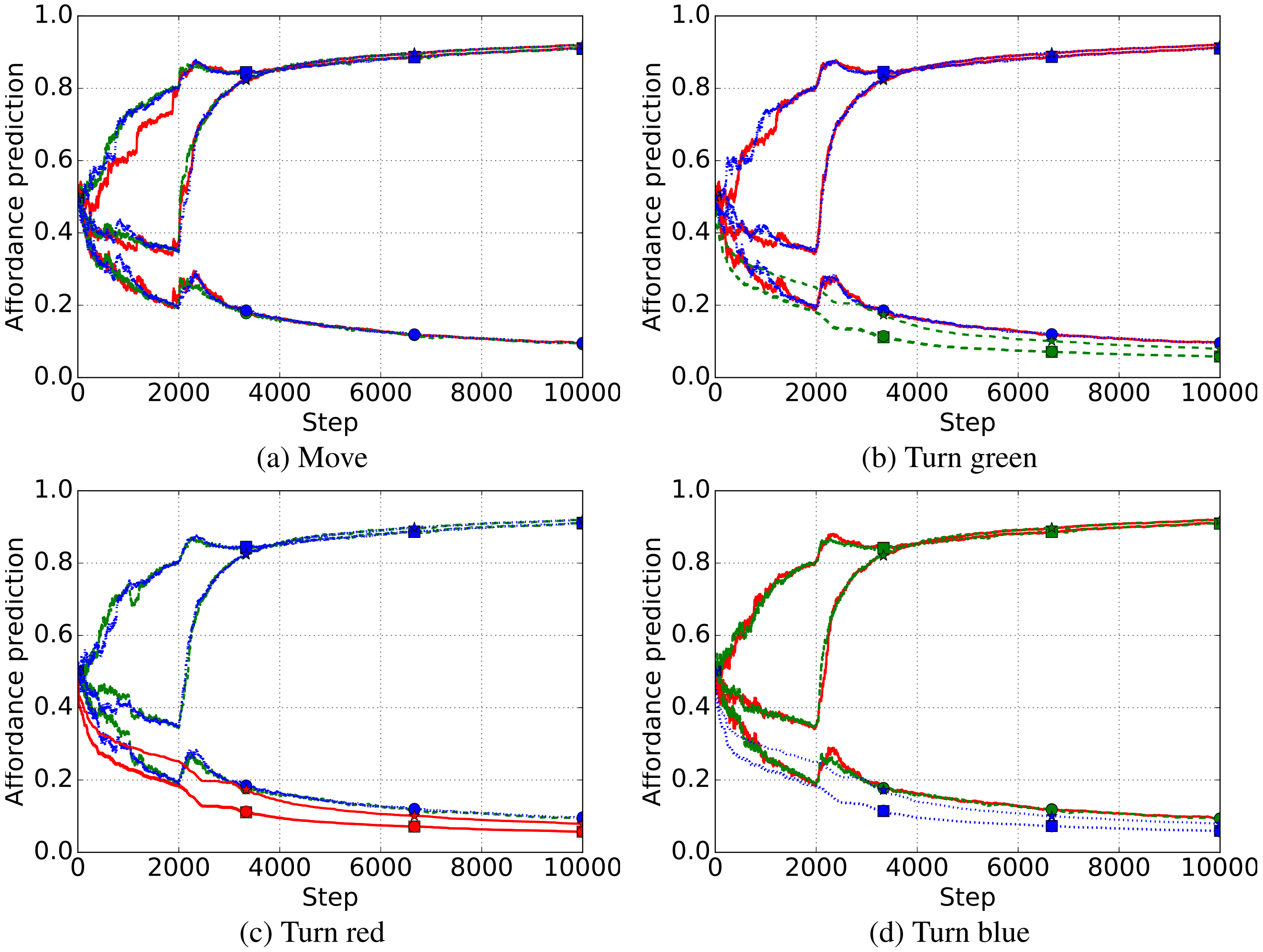


(a) Move (b) Turn green (c) Turn red (d) Turn blue

**Figure 1.** IGN model average affordance predictions in the second late objects experiment intrinsic phase, for all four actions (a, b, c, d) on all nine objects (red, green and blue; squares marked as squares, circles marked as circles and rectangles marked as stars).

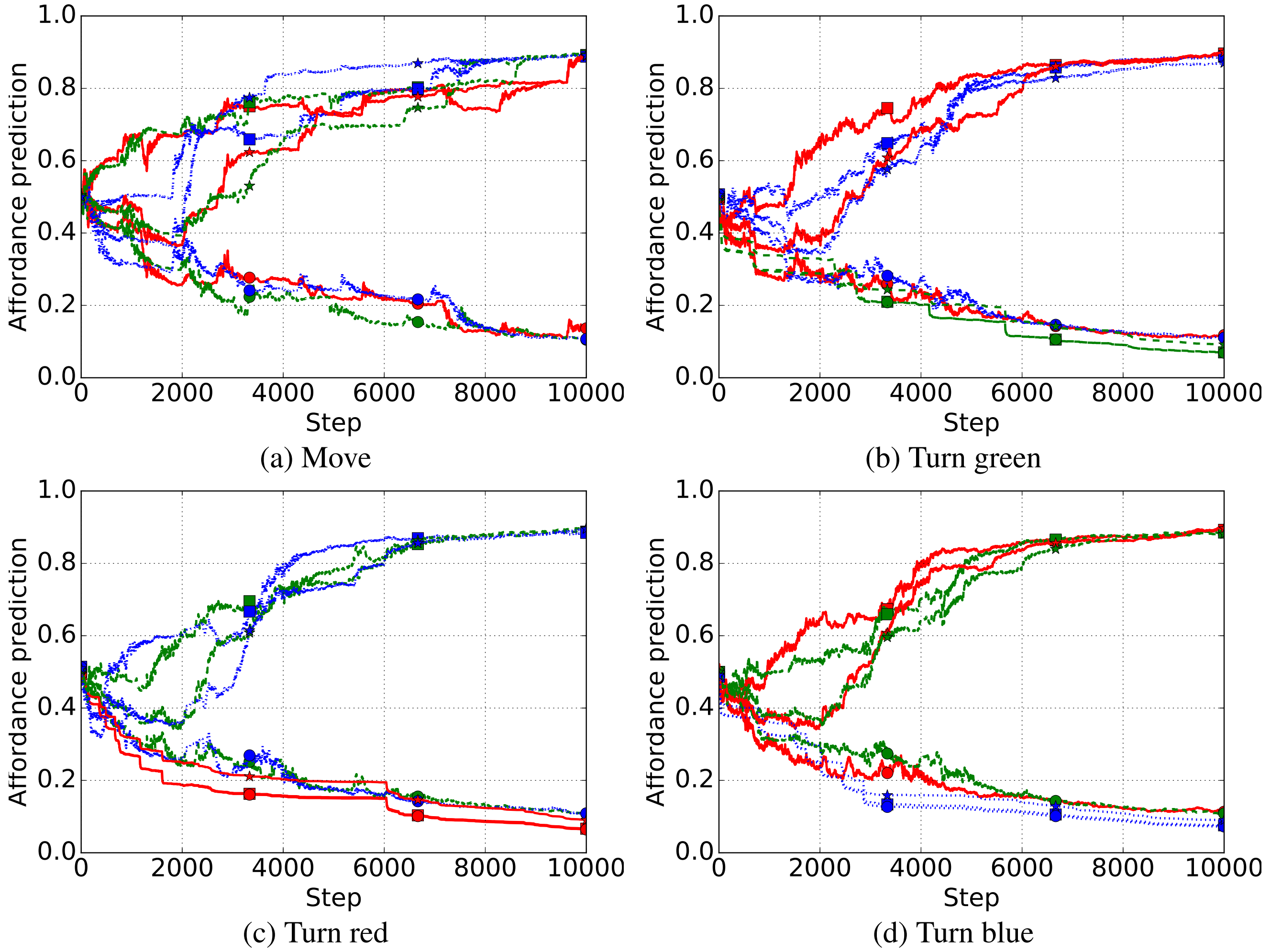


**Figure 2.** IMP model average affordance predictions in the second late objects experiment intrinsic phase, for all four actions (a, b, c, d) on all nine objects (red, green and blue; squares marked as squares, circles marked as circles and rectangles marked as stars).

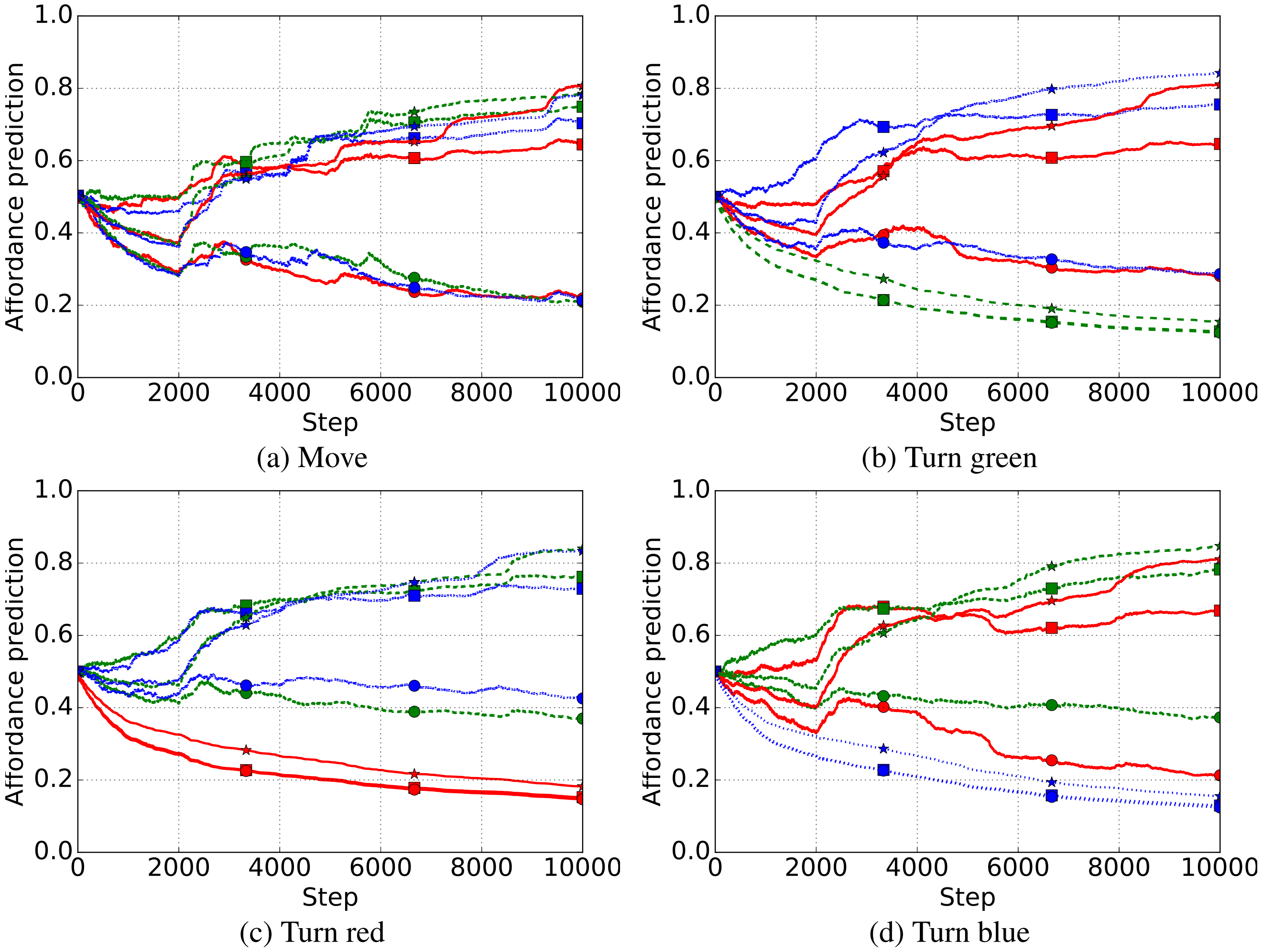


**Figure 3.** FIX model average affordance predictions in the second late objects experiment intrinsic phase, for all four actions (a, b, c, d) on all nine objects (red, green and blue; squares marked as squares, circles marked as circles and rectangles marked as stars).

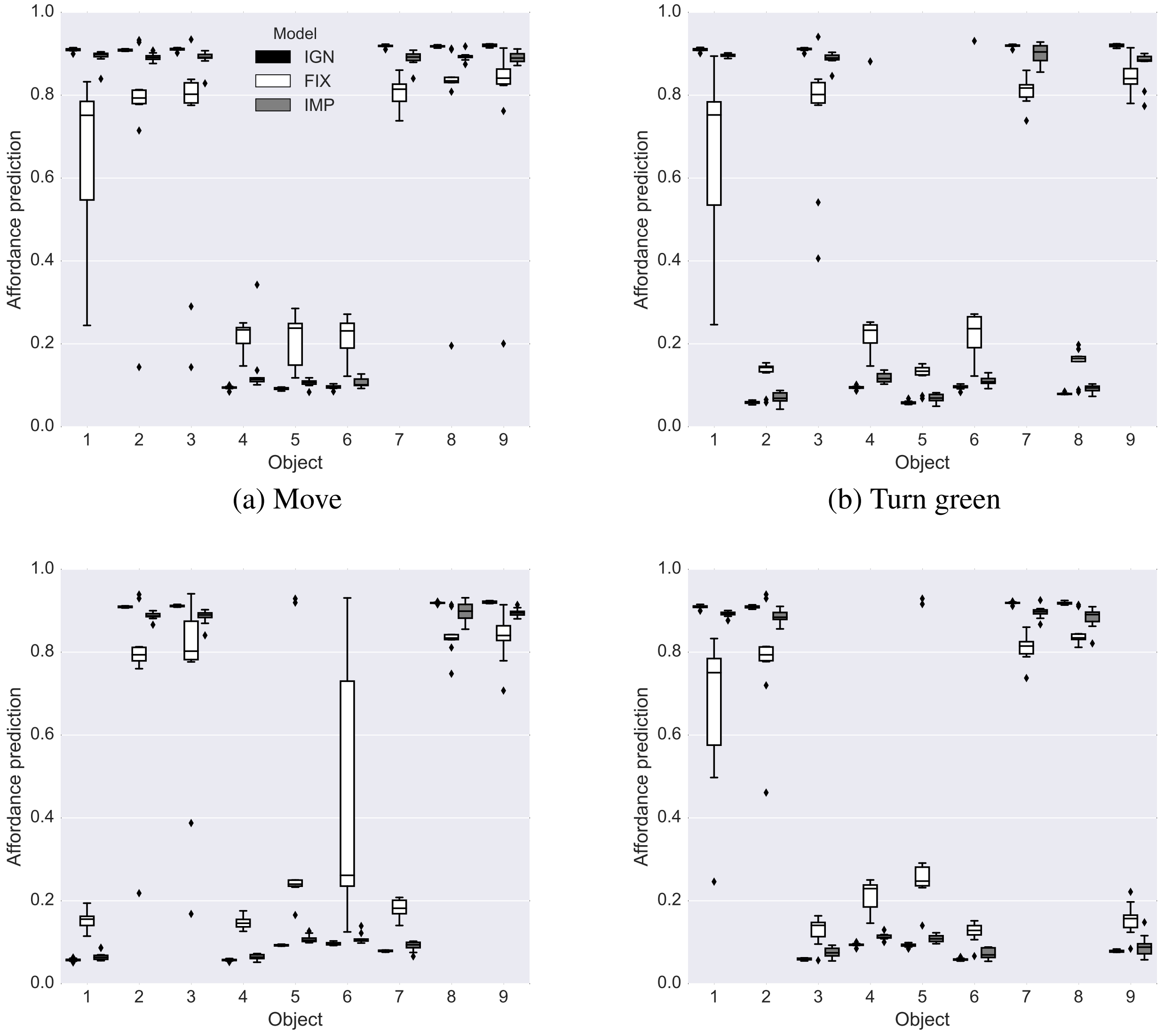


(a) Move (b) Turn green (c) Turn red (d) Turn blue

**Figure 4.** Second late objects test, affordance predictions for all four actions (a, b, c, d) on all nine objects for the 10 simulations after 10000 steps of intrinsic phase.

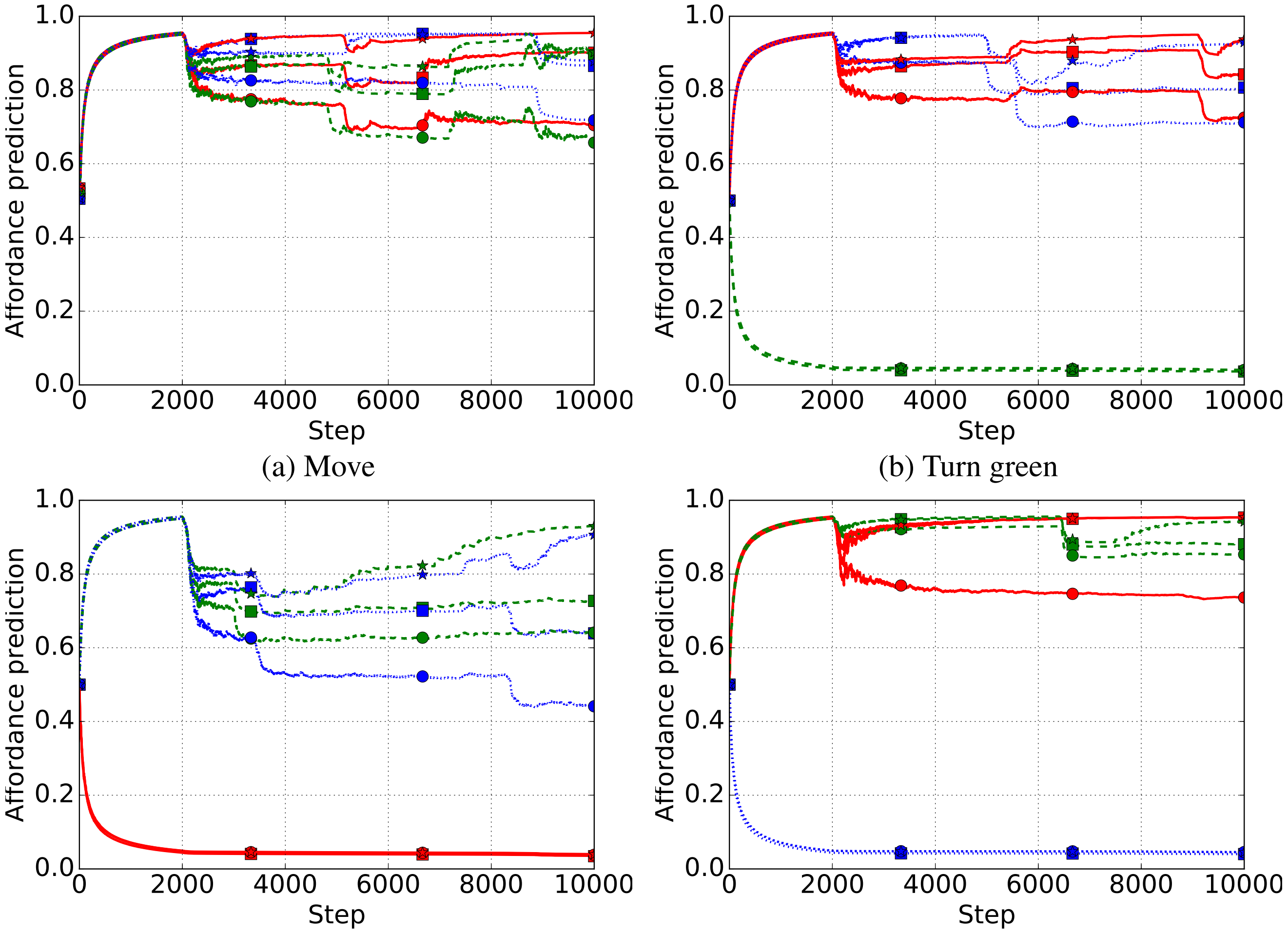


(a) Move (b) Turn green (c) Turn red (d) Turn blue

**Figure 5.** IGN model average affordance predictions in the third late objects experiment intrinsic phase, for all four actions (a, b, c, d) on all nine objects (red, green and blue; squares marked as squares, circles marked as circles and rectangles marked as stars).

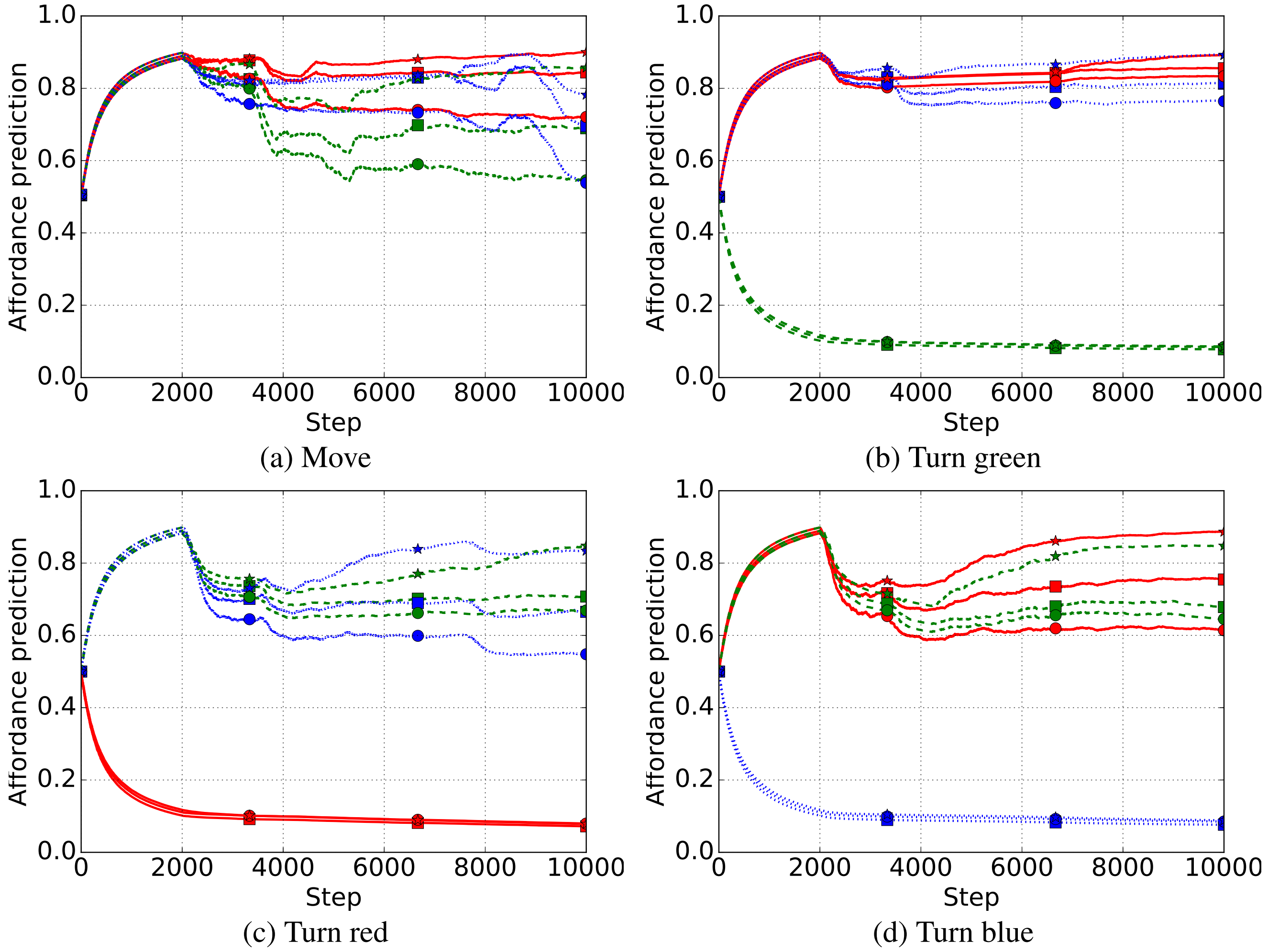


**Figure 6.** FIX model average affordance predictions in the third late objects experiment intrinsic phase, for all four actions (a, b, c, d) on all nine objects (red, green and blue; squares marked as squares, circles marked as circles and rectangles marked as stars).

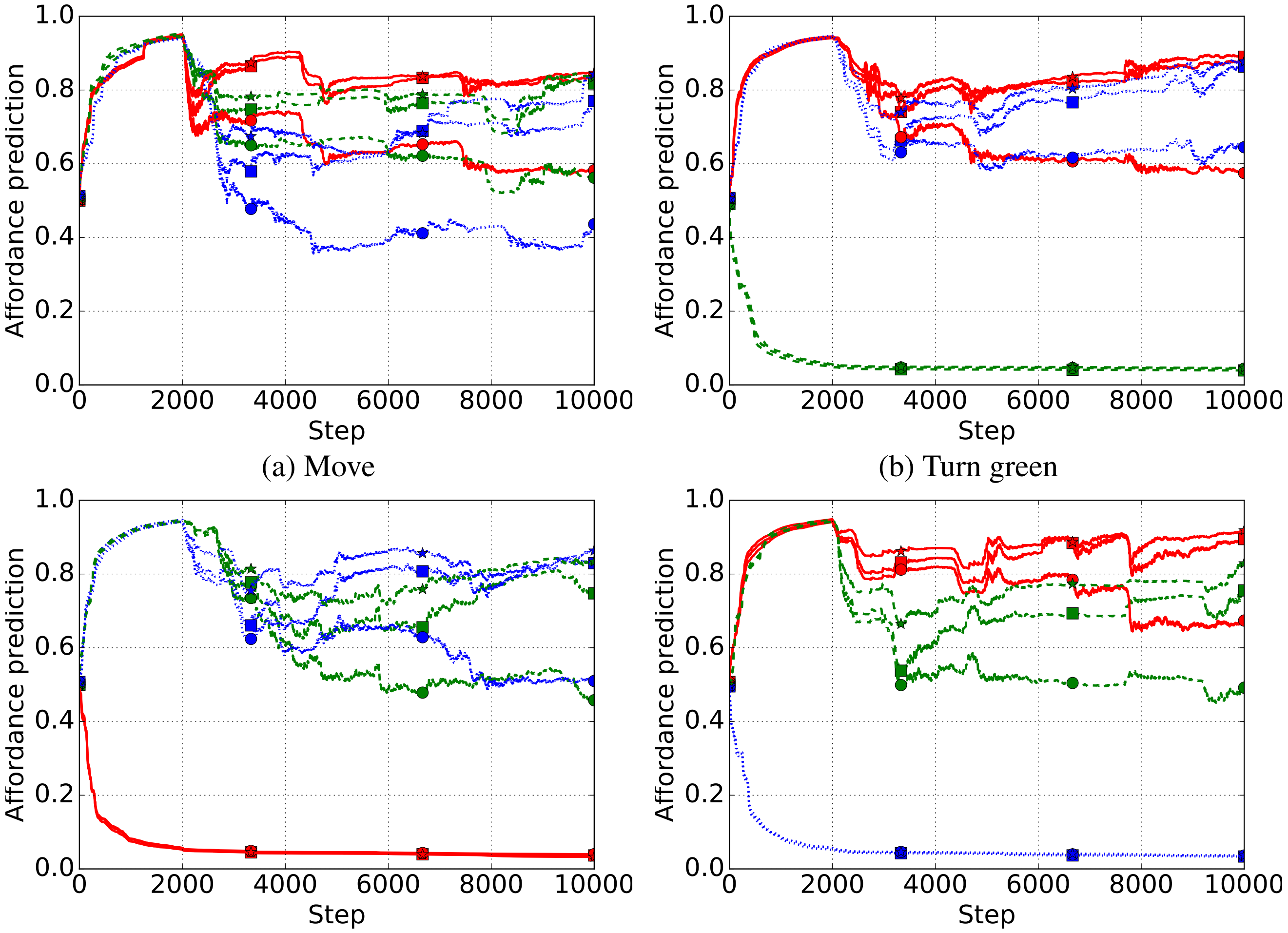


(a) Move (b) Turn green (c) Turn red (d) Turn blue

**Figure 7.** IMP model average affordance predictions in the third late objects experiment intrinsic phase, for all four actions (a, b, c, d) on all nine objects (red, green and blue; squares marked as squares, circles marked as circles and rectangles marked as stars).

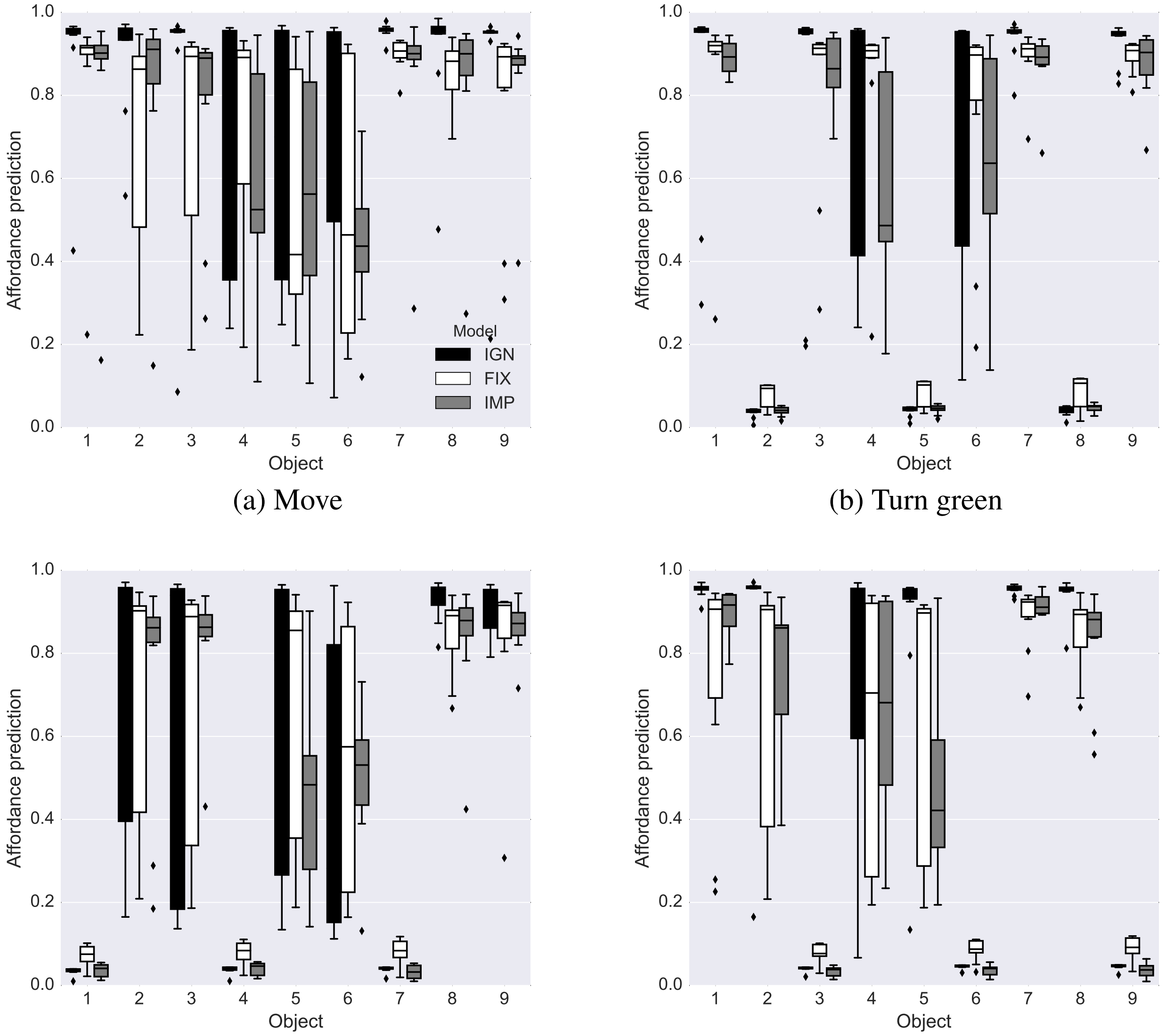


(a) Move (b) Turn green

(c) Turn red (d) Turn blue

**Figure 8.** Third late objects test, affordance predictions for all four actions (a, b, c, d) on all nine objects for the 10 simulations after 10000 steps of intrinsic phase.
